# PARP inhibition causes premature loss of cohesion in cancer cells

**DOI:** 10.18632/oncotarget.21879

**Published:** 2017-10-16

**Authors:** Eva Kukolj, Tanja Kaufmann, Amalie E. Dick, Robert Zeillinger, Daniel W. Gerlich, Dea Slade

**Affiliations:** ^1^ Max F. Perutz Laboratories, University of Vienna, Vienna Biocenter (VBC), Dr. Bohr-Gasse 9, Vienna, Austria; ^2^ Institute of Molecular Biotechnology of the Austrian Academy of Sciences (IMBA), Vienna Biocenter (VBC), Dr. Bohr-Gasse 3, Vienna, Austria; ^3^ Molecular Oncology Group, Department of Obstetrics and Gynecology, Medical University of Vienna, Vienna, Austria

**Keywords:** PARP inhibitors, mitosis, cohesion, PARP entrapment, live-cell imaging

## Abstract

Poly(ADP-ribose) polymerases (PARPs) regulate various aspects of cellular function including mitotic progression. Although PARP inhibitors have been undergoing various clinical trials and the PARP1/2 inhibitor olaparib was approved as monotherapy for BRCA-mutated ovarian cancer, their mode of action in killing tumour cells is not fully understood. We investigated the effect of PARP inhibition on mitosis in cancerous (cervical, ovary, breast and osteosarcoma) and non-cancerous cells by live-cell imaging. The clinically relevant inhibitor olaparib induced strong perturbations in mitosis, including problems with chromosome alignment at the metaphase plate, anaphase delay, and premature loss of cohesion (cohesion fatigue) after a prolonged metaphase arrest, resulting in sister chromatid scattering. PARP1 and PARP2 depletion suppressed the phenotype while PARP2 overexpression enhanced it, suggesting that olaparib-bound PARP1 and PARP2 rather than the lack of catalytic activity causes this phenotype. Olaparib-induced mitotic chromatid scattering was observed in various cancer cell lines with increased protein levels of PARP1 and PARP2, but not in non-cancer or cancer cell lines that expressed lower levels of PARP1 or PARP2. Interestingly, the sister chromatid scattering phenotype occurred only when olaparib was added during the S-phase preceding mitosis, suggesting that PARP1 and PARP2 entrapment at replication forks impairs sister chromatid cohesion. Clinically relevant DNA-damaging agents that impair replication progression such as topoisomerase inhibitors and cisplatin were also found to induce sister chromatid scattering and metaphase plate alignment problems, suggesting that these mitotic phenotypes are a common outcome of replication perturbation.

## INTRODUCTION

Poly(ADP-ribose) polymerases (PARPs) are enzymes important for diverse cellular processes ranging from transcriptional regulation and cell-cycle control, to chromatin dynamics, DNA repair, mitosis and cell death [1–3]. PARPs synthesize poly(ADP-ribose) (PAR) from NAD by attaching ADP-ribose units via glycosidic ribose-ribose bonds onto themselves (auto-modification) and other protein acceptors (hetero-modification) [4]. PAR―a short-lived post-translational modification―is an ideal mediator of dynamic cellular processes based on the formation of interaction scaffolds or the disruption of protein-protein and protein-DNA interactions [5]. More than 90% of cellular PAR is synthesized by PARP1 as the most abundant and the most highly active PARP [6].

Regulation of various DNA repair pathways such as single-strand break repair (SSBR), homologous recombination (HR) and non-homologous end joining remains the best studied role of PARP1 [7, 8]. Additionally, PARP1 promotes replication fork reversal and HR-dependent restart of stalled or collapsed replication forks [9–12]. PARP inhibition causes stalling or collapse of replication forks, resulting in lethal double-strand DNA breaks (DSBs) [13]. Replication blockage is presumably a consequence of entrapment and accumulation of inactive PARP1 on DNA by NAD-mimicking PARP inhibitors [13]. Sensitization of HR-deficient cancer cells by PARP inhibition has given rise to synthetic lethality approaches, whereby pharmacological inhibition of one DNA repair pathway coupled with genetic defects in another pathway causes lethality due to inability to repair damaged DNA [14]. In the first example of synthetic lethality induced by PARP inhibition, PARP1/2 inhibitor was shown to induce chromosomal instability, cell cycle arrest and apoptosis in breast cancer patients carrying heterozygous loss-of-function *BRCA* mutations [15, 16]. Another example of synthetic lethality between PARP1 inhibition and cohesin mutations further corroborates the importance of PARP1 for replication fork stability [17].

In addition to DNA repair, the roles of PARPs in the regulation of inflammatory mediators, cellular energetics, cell fate, gene transcription, ERK-mediated signalling and mitosis might underlie the susceptibility of cancer cells to PARP inhibition [18]. PARPs have distinct mitotic functions. PARP1 and PARP2 localize at centromeres and interact with centromeric proteins [19]. PARP1 is required for the maintenance of the spindle assembly checkpoint and post-mitotic checkpoint; its depletion or inhibition result in centrosome amplification and aneuploidy [20–22]. PARP1 knock-out mouse oocytes exhibit incomplete synapsis of homologous chromosomes, deficient sister chromatid cohesion during metaphase II and failure to maintain metaphase arrest due to lack of centromeric recruitment of the mitotic checkpoint protein BUB3 [23]. The E3 ubiquitin ligase CHFR (checkpoint with FHA and RING finger domains) regulates the mitotic checkpoint via PARP1 ubiquitination and degradation during mitotic stress, resulting in cell cycle arrest in prophase [24]. Tankyrase (PARP5) has also been implicated in mitotic regulation; it is found around the pericentriolar matrix of mitotic chromosomes and was shown to regulate spindle assembly [25, 26] together with PARP3 [27].

Olaparib is the only PARP1/2 inhibitor approved for treatment of pretreated or platinum sensitive ovarian cancer associated with defective BRCA1/2 genes. Talazoparib is the most potent PARP1/2 inhibitor developed to date, exerting its cytotoxicity by PARP trapping rather than catalytic inhibition [28]. The catalytic inhibitory effect of talazoparib is comparable to olaparib; nevertheless, it is 100-fold more potent at trapping PARP-DNA complexes [28]. Veliparib is among the least potent PARP1/2 inhibitors with weak catalytic inhibition and low PARP trapping efficiency [13]. All three inhibitors are currently undergoing various clinical trials.

Considering the multiple roles of PARP in mitosis, we investigated the effect of PARP inhibition on mitotic progression by live-cell imaging. PARP1/2 inhibition with olaparib, talazoparib or veliparib induced metaphase arrest and sister chromatid scattering in HeLa cells, leading to cell death. Chromatid scattering in mitosis was caused by premature loss of cohesion in interphase cells whereby olaparib treatment caused a two-fold increase in sister chromatid distance. Premature loss of cohesion occurred when olaparib was added already during S-phase, suggesting that replication fork blockage due to PARP entrapment leads to loss of cohesion and subsequent defects in mitosis. Premature loss of cohesion was also observed in cancer cell lines of cervical, breast and osteosarcoma origin that exhibit S-phase stalling upon olaparib treatment. The severity of this mitotic phenotype across different cell lines correlated with PARP1 and PARP2 protein levels, was rescued by PARP1 or PARP2 depletion and exacerbated by PARP2 overexpression. Similar mitotic phenotypes were also found upon treatment with DNA-damaging agents that cause S-phase stalling such as topoisomerase inhibitors (camptothecin, etoposide) and cisplatin, suggesting that death by mitotic failure is a general phenomenon of perturbed replication.

## RESULTS

### Olaparib causes anaphase delay and chromatid scattering in metaphase-arrested cells

**Figure 1:**
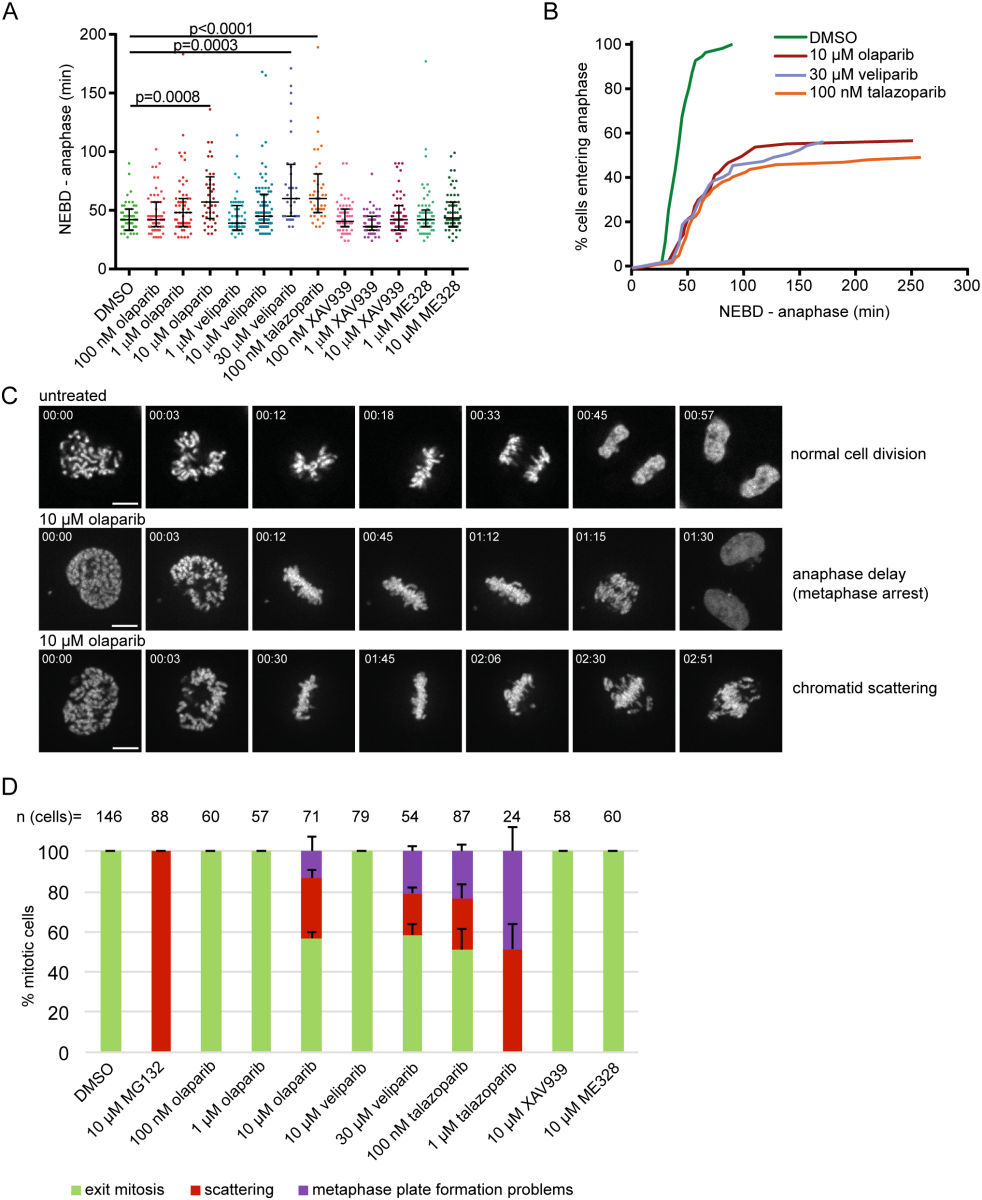
PARP1/2 inhibition causes anaphase delay and chromatid scattering (premature loss of cohesion) in HeLa cells **(A)** Duration of NEBD-anaphase was analysed by live imaging of HeLa cells stably expressing H2B-mCherry EGFP-securin treated with PARP inhibitors for 30 h. Each dot represents one cell. **(B)** Cumulative frequency distribution representing both anaphase delay and inability to enter anaphase in cells treated with indicated concentrations of PARP inhibitors. **(C)** H2B-mCherry stills of representative mitotic phenotypes. Scale bar=10 μm. **(D)** Percentage of different mitotic phenotypes observed after treatment with PARP inhibitors for 30-37 h and MG132 as a positive control for 1 h.

In order to investigate the effect of PARP inhibition on mitosis, we performed live-cell imaging of HeLa cells stably expressing H2B-mCherry together with securin-EGFP [29] treated with olaparib (AZD2281, Ku-0059436) [30], talazoparib (BMN 673) [31] or veliparib (ABT-888) [32] as PARP1/2 inhibitors, XAV-939 as a tankyrase1/2 (PARP5a/b) inhibitor [33] and ME328 as a PARP3 inhibitor (Figure 1A) [34]. Of the five tested inhibitors applied at different concentrations for 30 h, only PARP1/2 inhibitors caused anaphase delay measured as the time required for the cells to progress from nuclear envelope breakdown (NEBD) to anaphase (Figure 1A, B). The median NEBD-anaphase duration was extended from 42 min in DMSO-treated control cells to 57 min for 10 μM olaparib-treated cells, to 60 min for 30 μM veliparib and to 60 min for 100 nM talazoparib (Figure 1A, B). 40-50% of inhibitor-treated mitotic cells failed to enter anaphase due to metaphase plate formation problems (inability to align chromosomes on the metaphase plate) or chromatid scattering after correct metaphase plate formation (Figure 1B–1D).

Chromatid scattering was initially described as a result of prolonged metaphase in cells treated with the proteasome inhibitor MG132 or depleted of the anaphase promoting complex/cyclosome (APC/C) activator Cdc20, and thought to result from premature loss of cohesion (cohesion fatigue) [35, 36]. MG132, as a positive control, induced chromatid scattering in all mitotic cells after 1 h treatment (Figure 1D). Chromatid scattering was observed in 30±4% of cells treated with 10 μM olaparib for 30-37 h, 21±3% of cells treated with 30 μM veliparib and 26±7% of cells treated with 100 nM talazoparib (Figure 1D). These cells were arrested in a metaphase-like state as revealed by high levels of cyclin B and securin (Supplementary Figure 1). Unless cyclin B and securin are ubiquitinated by the APC leading to proteosomal degradation, separase-mediated cleavage of centromeric cohesion cannot promote metaphase to anaphase transition [37]. XAV-939 or ME328 did not induce the scattering phenotype (Figure 1D).

### Chromatid scattering correlates with the efficiency of inhibition and S-phase stalling induced by PARP inhibitors

We next examined whether chromatid scattering is linked with the efficiency of PARP1 inhibition and its effect on cell cycle progression (Supplementary Figure 2). 10 μM olaparib, 30 μM veliparib and 100 nM talazoparib efficiently inhibited PARP1 auto-PARylation as determined by Western blotting with an anti-PAR antibody (>90% reduction after 24 h compared to the DMSO-treated cells) (Supplementary Figure 2A). Although *in vitro* assays determined 1.4 nM and 12.3 nM as IC_50_ values of olaparib towards PARP1 and PARP2 [38], we found that olaparib has to be used at a much higher concentration in order to inhibit PARP1 in HeLa cells (Supplementary Figure 2A). However, the concentration of 10 μM olaparib that showed inhibition of PARP1 activity, anaphase delay and scattering is still significantly below the concentration of olaparib used in clinical trials (peak plasma concentration=24 μM) [39]. Our data in Supplementary Figure 2A confirm that talazoparib (IC_50_ values of 1.1 and 4.1 nM for PARP1 and PARP2, respectively) is a more potent inhibitor than olaparib, whereas veliparib (IC_50_ values of 3.3 and 17.5 nM for PARP1 and PARP2, respectively) is a weaker inhibitor [38]. Flow cytometry analysis confirmed that ≥1 μM olaparib, ≥100 nM talazoparib and ≥30 μM veliparib cause G2/M arrest [15, 40] (Supplementary Figure 2B). Depending on the incubation time, olaparib caused either stalling in S-phase and G2/M arrest (12 h incubation) or mainly G2/M arrest (28 and 32 h of incubation) (Supplementary Figure 2C). Neither XAV-939 nor ME328 had an effect on cell cycle progression (Supplementary Figure 2B).

These results indicate that the concentrations of olaparib, talazoparib and veliparib that induce chromatid scattering strongly inhibit PARP1 activity and perturb S-phase and G2 progression. The strong S-phase stalling is presumably due to the trapping of PARP1 and PARP2 on DNA by these inhibitors [13, 28]. The inhibitors differ in their efficiency of catalytic inhibition, S-phase stalling and chromatid scattering, with talazoparib>olaparib>veliparib. Taken together, our data reveal that in addition to S-phase stalling, G2/M arrest and anaphase delay, prolonged olaparib, talazoparib or veliparib treatment induce chromatid scattering in HeLa cells.

### PARP silencing does not cause chromatid scattering found after PARP inhibition

PARP inhibition and depletion of PARP protein does not always yield the same phenotypes. PARP inhibition is more effective in inducing apoptosis in BRCA-deficient cells compared to PARP knock-down [16]; wild-type cells treated with olaparib are more sensitive to MMS than PARP1 knock-out cells [13]; PARP inhibition impairs SSB repair to a greater extent than PARP depletion [41, 42]; the level of DSBs is higher in olaparib-treated than PARP-depleted cells under basal conditions [13]; PARP inhibition was shown to cause S-phase stalling whereas PARP silencing had no effect [13]. To test whether depleting PARP causes the same mitotic phenotypes as PARP inhibition, we started imaging cells 24 h after depleting individual PARP members by three different siRNAs and analysed the cells 41-58 h after siRNA transfection (Supplementary Figure 3 and Supplementary Table 1). Anaphase delay was observed for only two siRNAs targeting PARP1, one siRNA targeting PARP2 and two siRNAs targeting PARP3 (Supplementary Figure 3A-C) (siControl: 33 min; siPARP1: 42 min, 39 min; siPARP2: 57 min; siPARP3: 58 min, 54 min), which suggests that it is an off-target effect. Silencing tankyrase 1 did not cause anaphase delay (Supplementary Figure 3D). None of the siRNAs caused scattering (Supplementary Figure 3E). This suggests that entrapment of inactive PARP1/2 at replication forks is likely to be the cause of chromatid scattering observed after PARP1/2 inhibition.

### Chromatid scattering is caused by specific inhibition of PARP1 and PARP2 by olaparib

**Figure 2:**
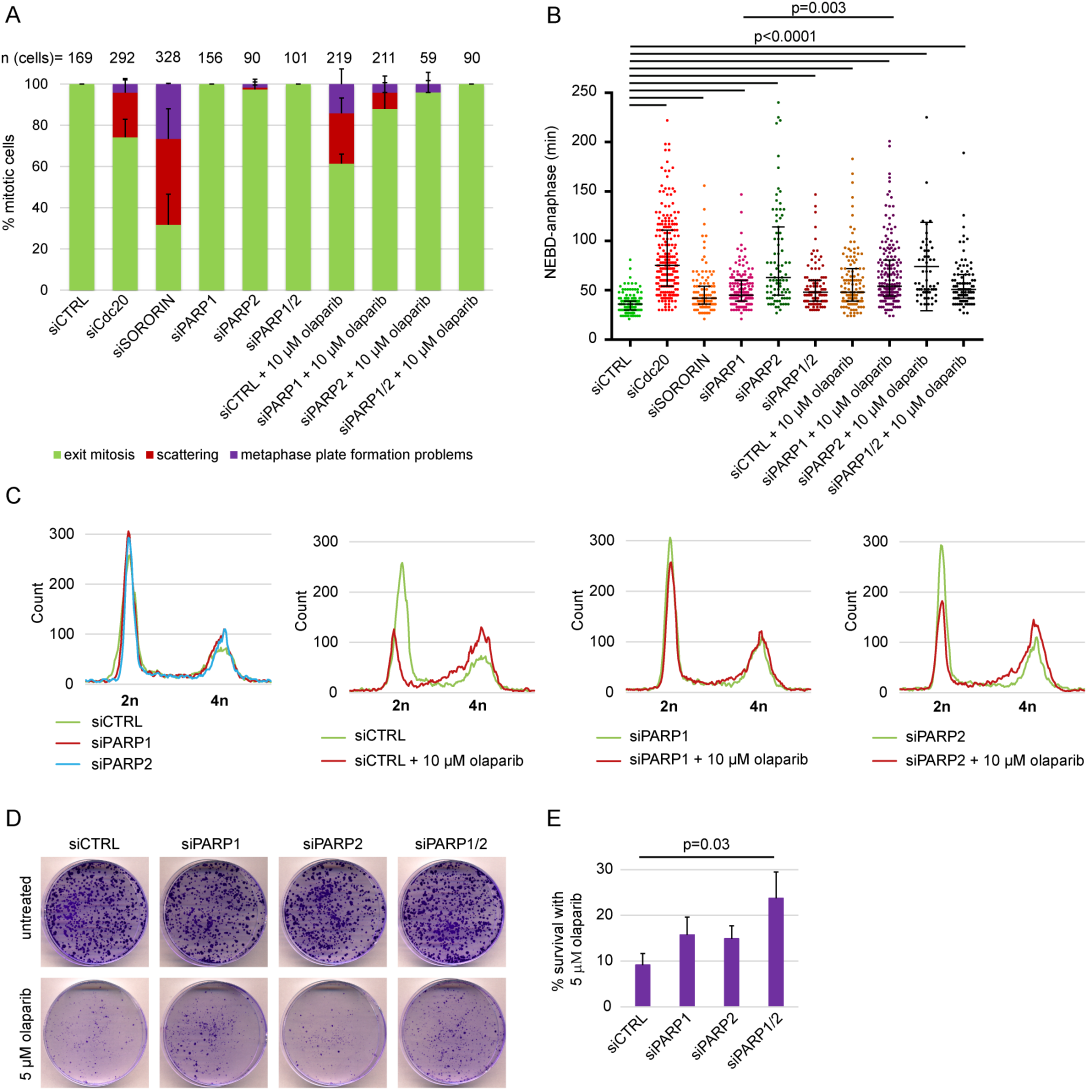
PARP1 and PARP2 depletion rescue olaparib-induced scattering, S-phase stalling and cytotoxicity but not anaphase delay in HeLa **(A)** Percentage of different mitotic phenotypes, **(B)** NEBD-anaphase duration, **(C)** flow cytometry analysis and **(D-E)** cell survival after concomitant treatment with olaparib and siPARP1/2 compared to single treatments. siPARP1 E and siPARP2 E were used. siCdc20 and siSORORIN were used as positive controls for live imaging and the cells were analysed 48-68 h after siRNA transfection. Imaged cells and FACS samples were analysed after 48 h of PARP silencing and 30 h of PARP inhibition. Cell survival was determined using colony formation assay 14 days after seeding. Cells were seeded 24 h after transfection with siRNAs and olaparib was added at the time of seeding.

PARP inhibition was previously shown to be more effective than PARP depletion and the cytotoxic effects of olaparib were directly linked with PARP1 rather than off-targets [13]. We also tested whether the scattering phenotype caused by PARP inhibition―but not depletion―is a specific or off-target effect of PARP inhibition (Figure 2). PARP1 depletion by RNAi reduced the degree of olaparib-induced scattering from 24±8 to 8±5% (Figure 2A). Both PARP2 and combined PARP1/2 depletion completely rescued olaparib-induced scattering (Figure 2A). This indicates that the scattering phenotype is caused by olaparib-inhibited PARP1 and PARP2, rather than an off-target inhibition by olaparib. Neither PARP1 nor PARP2 depletion rescued anaphase delay induced by olaparib, indicating that anaphase delay is not due to PARP entrapment (Figure 2B). Flow cytometry analysis additionally showed that PARP1 depletion in olaparib-treated cells reversed S-phase stalling and G2/M arrest, while PARP2 depletion had a partial effect (Figure 2C). Importantly, concomitant PARP1 and PARP2 depletion increased survival of HeLa cells exposed to 5 μM olaparib for 14 days from 9.2±2.5% to 24.3±5.7% according to colony formation assay (Figure 2D, E).

**Figure 3:**
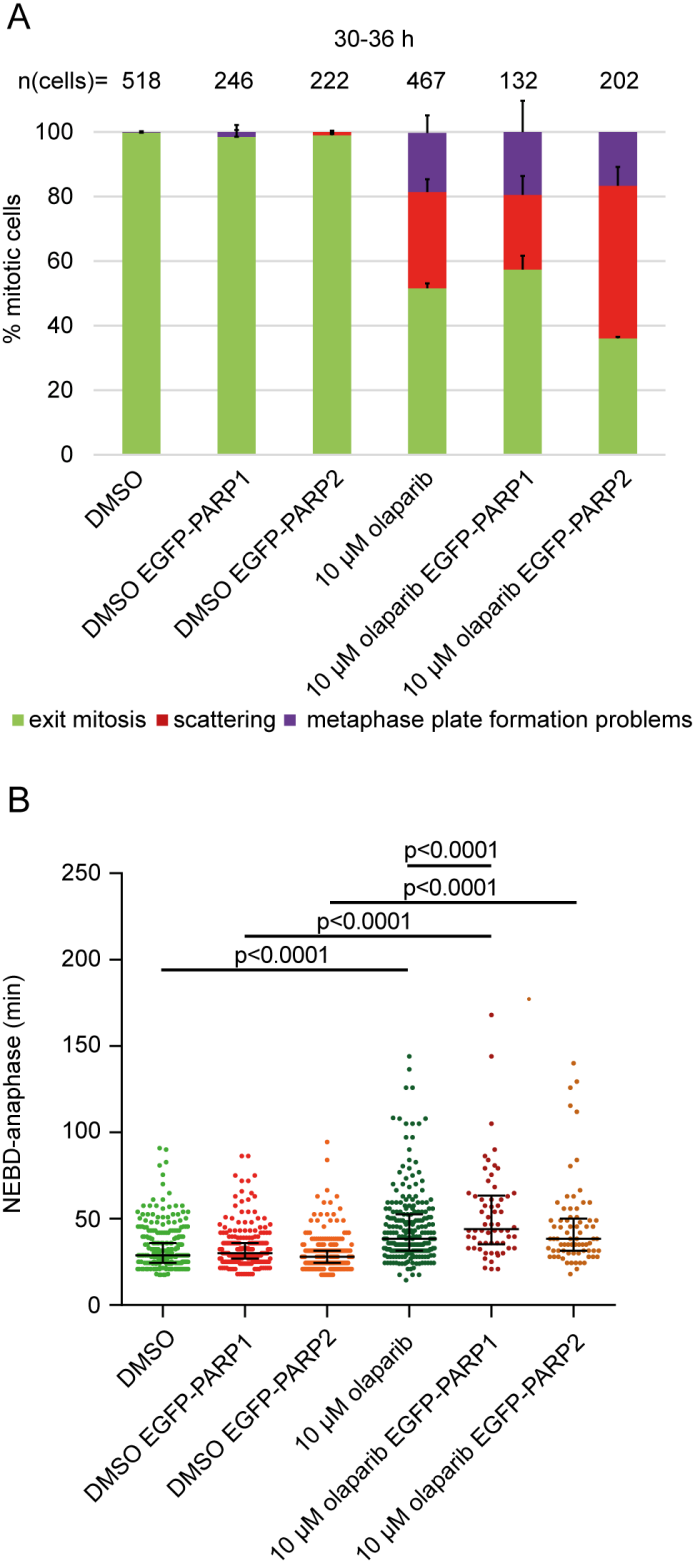
PARP2 overexpression exacerbates olaparib-induced scattering **(A)** Percentage of different mitotic phenotypes and **(B)** NEBD-anaphase duration in EGFP-PARP1 or EGFP-PARP2-overexpressing HeLa cells imaged 30-36 h after treatment with 10 μM olaparib.

Given that PARP1/2 silencing rescued olaparib-induced scattering, we also tested whether PARP1/2 overexpression can exacerbate the phenotype (Figure 3). We generated EGFP-PARP1 or EGFP-PARP2 overexpressing HeLa cells and performed live-cell imaging. PARP1 overexpression had no effect on scattering but slightly increased olaparib-induced anaphase delay only in the presence of olaparib (from 35 to 38.5 min); PARP2 increased the degree of scattering from 34±4% to 47±6% (p=0.037), without affecting anaphase delay (Figure 3). Taken together, our data indicate that (i) chromatid scattering is a specific outcome of olaparib-mediated PARP1 and PARP2 inhibition, (ii) PARP2 inhibition has a stronger effect on scattering, and (iii) olaparib cytotoxicity can be partly rescued by depleting PARP1 and PARP2.

### Chromatid scattering is caused by the S-phase effects of olaparib

**Figure 4:**
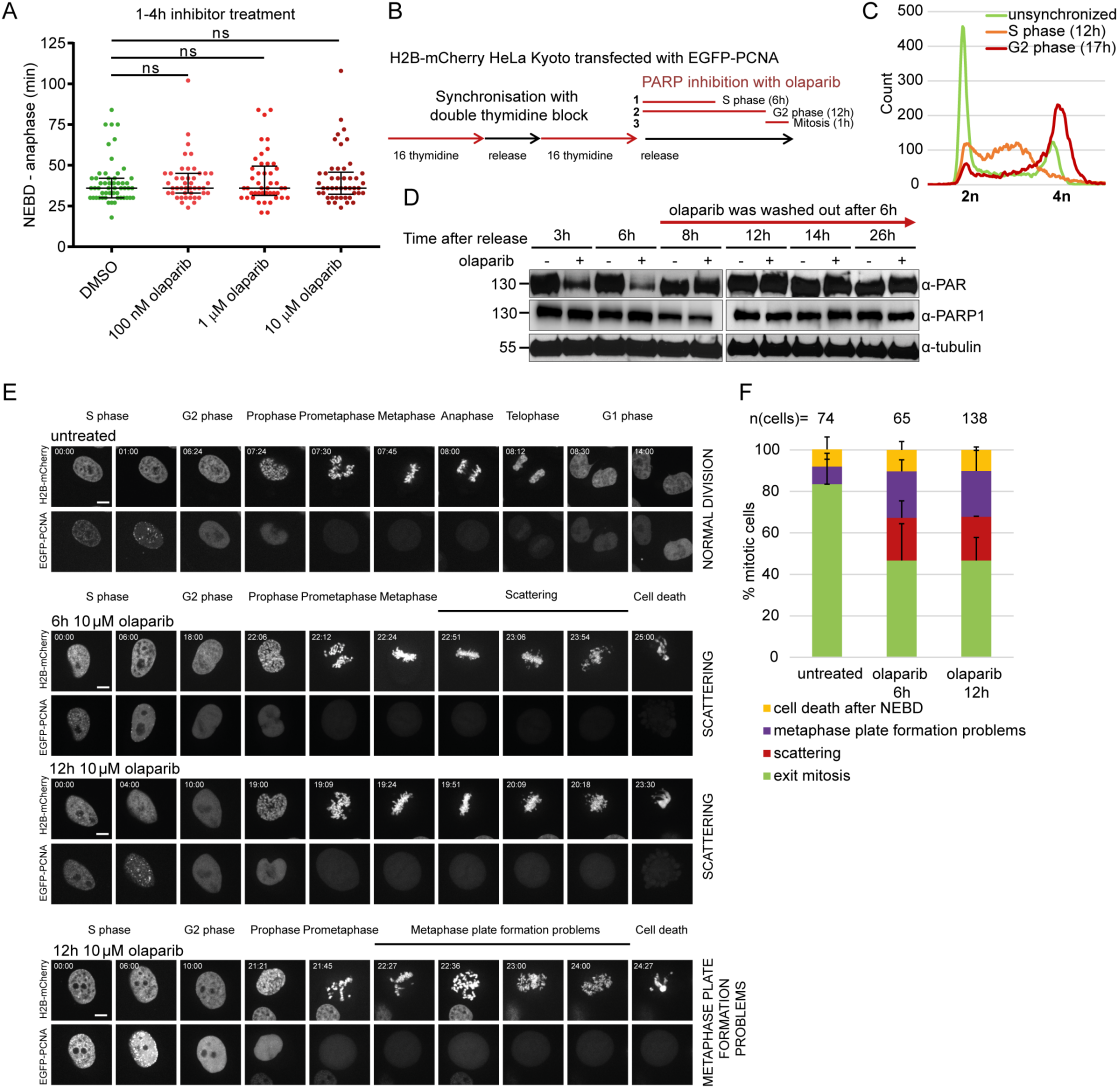
Premature loss of cohesion caused by olaparib is due to PARP inhibition in S-phase **(A)** Duration of NEBD-anaphase analysed by live-cell imaging in HeLa cells stably expressing H2B-mCherry EGFP-securin treated for 1-4 h with indicated concentrations of olaparib. **(B)** Experimental workflow for olaparib exposure during different stages of the cell cycle. HeLa H2B-mCherry cells were transfected with EGFP-PCNA and synchronized by double thymidine block as shown with FACS profiles under **(C)**. Olaparib was added ‘1’ during S-phase, ‘2’ during S and G2 phase, ‘3’ just before NEBD. **(D)** Western blot analysis showing efficient removal of olaparib at the end of S-phase. **(E)** Stills from spinning disc imaging and **(F)** quantification of representative mitotic phenotypes. Scale bar=10 μm.

Olaparib did not perturb mitotic progression if added just before the onset of mitosis (Figure 4A), as measured by scoring cells that entered mitosis 1-4 h upon olaparib treatment. This suggests that defects from earlier cell cycle stages gave rise to the observed mitotic phenotypes. To test this, H2B-mCherry HeLa cells transfected with EGFP-PCNA were synchronized by double thymidine block (Figure 4B, C). Olaparib was added (1) immediately after the release into S-phase and washed out after 6 h (estimated S-phase duration in HeLa cells according to EGFP-PCNA foci); (2) immediately after the release into S-phase and washed out before mitotic entry; (3) 30 min before mitosis (NEBD considered as mitotic entry) (Figure 4B). Olaparib was efficiently washed out as judged by the restoration of PARP1 auto-PARylation within two hours of olaparib removal (Figure 4D). Live-cell imaging on a spinning disc setup enabled us to distinguish with confidence between the sister chromatid scattering phenotype and the metaphase plate formation problems (Figure 4E). The presence of olaparib only during S-phase (6 h treatment) was sufficient to induce chromatid scattering and metaphase plate formation problems in 21±8% and 22±5% of mitotic cells, respectively (Figure 4E, F). Both phenotypes resulted in cell death (Figure 4E). Cell death occurring after NEBD was observed in additional 11% of the analysed mitotic population as well as in 8% of untreated cells, and may be due to anaphase-related segregation defects, thymidine-induced damage or physical causes (e.g., phototoxicity during imaging) (Figure 4F). PARP inhibition during S-phase thus causes subsequent perturbations during mitosis leading to cell death.

### Olaparib directly induces premature loss of cohesion

**Figure 5:**
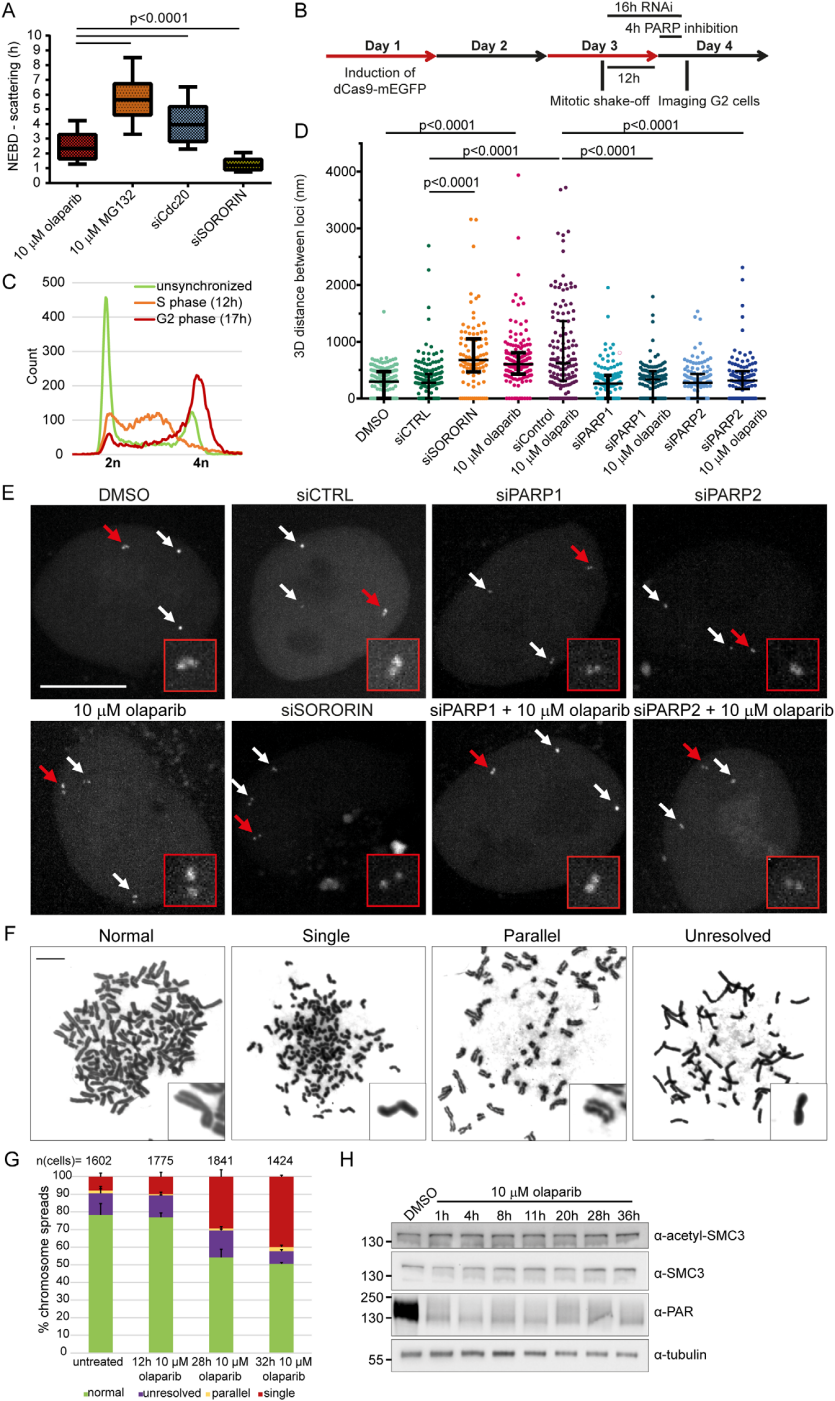
Scattering is a result of premature loss of cohesion caused by olaparib **(A)** Comparison of NEBD-scattering duration induced by various treatments. Cells were treated with 10 μM olaparib for 24 h and 10 μM MG132 for 30 min. Cdc20 and sororin were depleted for 24 h with RNAi. Number of cells analysed per condition: n (olaparib) = 44, n (MG132) = 88, n (siCdc20) = 94, n (siSororin) = 63. **(B)** Experimental workflow for measuring sister chromatid distances. dCas9-mEGFP targeting Muc4 loci is induced, followed by mitotic shake-off, (i) 12 h growth and PARP inhibition for 4 h, or (ii) RNAi for 16 h before imaging of G2 cells. **(C)** FACS profiles showing synchronization of Muc4-labeled HeLa cells in S and G2 phase. **(D)** 3D distance between sister chromatids at Muc4 loci measured after live-cell imaging of PARP-inhibited or PARP/sororin-depleted cells. **(E)** Representative images quantified under (D). Scale bar=10 μm. **(F)** Representative images and **(G)** analysis of phenotypes revealed by Giemsa-stained chromosome spreads after addition of 10 μM olaparib. Scale bar=10 μm. **(H)** Olaparib does not affect SMC3 acetylation levels. Extracts from HeLa cells treated with 10 μM olaparib for various times were analysed by Western blotting.

Premature loss of cohesion was previously described as a common outcome of metaphase arrest, induced by agents that are not directly affecting cohesion (e.g., MG132, siCdc20) [36]. To test whether olaparib induces premature sister chromatid separation directly by weakening sister chromatid cohesion, we compared the timing of scattering after olaparib treatment to other treatments previously shown to induce chromatid scattering (Figure 5A). In addition to MG132 and siCdc20, partial RNAi depletion of sororin, which is required for cohesion establishment in S-phase, was also shown to induce chromatid scattering (Supplementary Figure 4) [43, 44]. Cells arrested in metaphase with MG132 or siCdc20 exhibited chromatid scattering only after 4 h; however, siSororin rapidly induced scattering (1.2 h after NEBD), while 10 μM olaparib exhibited intermediate kinetics (2.4 h after NEBD) (Figure 5A). This indicates that olaparib treatment may directly weaken chromosome cohesion.

To directly measure the effect of olaparib on sister chromatid distances during interphase, we visualized a genomic locus (Muc4) in live HeLa cells by stably expressing dCas9-mEGFP and guide RNAs targeting 3^rd^ exon of the Muc4 gene (hg19 Chr3: 195506180-195510888), as previously reported [45]. We synchronized cells by mitotic shake-off and imaged the labeled loci 16 h later, when cells were in G2 (Figure 5B, C). 64% of the labelled loci appeared as doublet dots (Figure 5D, E), consistent with a separation of the two replicated sister loci by a distance larger than the resolution limit of the confocal microscopy. The median distance between separated sister loci in control cells was 275 nm (n=195) and it increased in sororin-depleted cells to 670 nm (n=94) (Figure 5D, E). A 4 h-treatment of S-phase cells with 10 μM olaparib (siControl + 10 μM olaparib) also substantially increased the distance between sister loci to 617 nm (n=136), whereas siPARP1 and siPARP2 had no effect (Figure 5B–5E).

This confirms that S-phase PARP1/2 inhibition by olaparib directly weakens sister chromatid cohesion in interphase cells ultimately resulting in sister chromatid scattering in metaphase cells. Importantly, siPARP1 and siPARP2 rescued the effect of olaparib, confirming that olaparib-induced sister chromatid separation is caused by on-target inhibition of PARP1/2 (Figure 5B–5E).

In addition, we analysed chromosome spreads from untreated and olaparib-treated HeLa cells collected by mitotic shake-off (Figure 5F, G). 7.7±1.9% of untreated spreads contained single chromatids (Figure 5F, G). These single chromatids may correspond to anaphase stages, depending on how the cells fell on the glass slide. 10 μM olaparib gave rise to 29.3±3.9% and 39.9±0.7% of spreads with single chromatids at 28 or 32 h after treatment, respectively (Figure 5F, G). This corresponds well to the percentage of cells exhibiting chromatid scattering (30±4% for 10 μM olaparib after 30-37 h) (Figure 1D).

Acetylation of SMC3 was shown to mediate cohesion establishment in S-phase by promoting sororin recruitment to the cohesin complex [43]. Thus we tested whether olaparib impairs cohesion establishment by examining SMC3 acetylation in olaparib-treated cells (Figure 5H). Acetylation of SMC3 did not change upon olaparib treatment, which suggests that olaparib does not impair cohesion establishment but instead causes loss of cohesion. Cohesion defects caused by depletion of spliceosome subunits were shown not to affect SMC3 acetylation either [46, 47], confirming that loss of cohesion can occur despite steady-state levels of SMC3 acetylation.

Taken together, our data provide comprehensive evidence that the olaparib-induced scattering phenotype results from premature loss of sister chromatid cohesion in replicating cells.

### Olaparib causes chromatid scattering in several cancer cell lines

The above characterization of olaparib effects on mitotic progression was performed on HeLa cells as a commonly used cancer cell line of cervical origin. We extended our characterization to a set of non-cancerous cell lines (human mammary epithelial, HME1 [48]; retinal pigment epithelial, RPE1 [36]) and cells of cancerous origin from cervix (C33-A, SiHa), ovary (TOV-21G), breast (MDA-MB-468 [49] and BT-549) and osteosarcoma (U2OS [50]) (Supplementary Table 2) and visualized chromosomes in live cells with the non-toxic SiR-Hoechst DNA dye [51]. Anaphase delay and chromatid scattering in HeLa SiR-Hoechst-labelled cells treated with olaparib were comparable to HeLa H2B-mCherry cells (Supplementary Figure 5), validating that SiR-Hoechst does not interfere with our assay.

**Figure 6:**
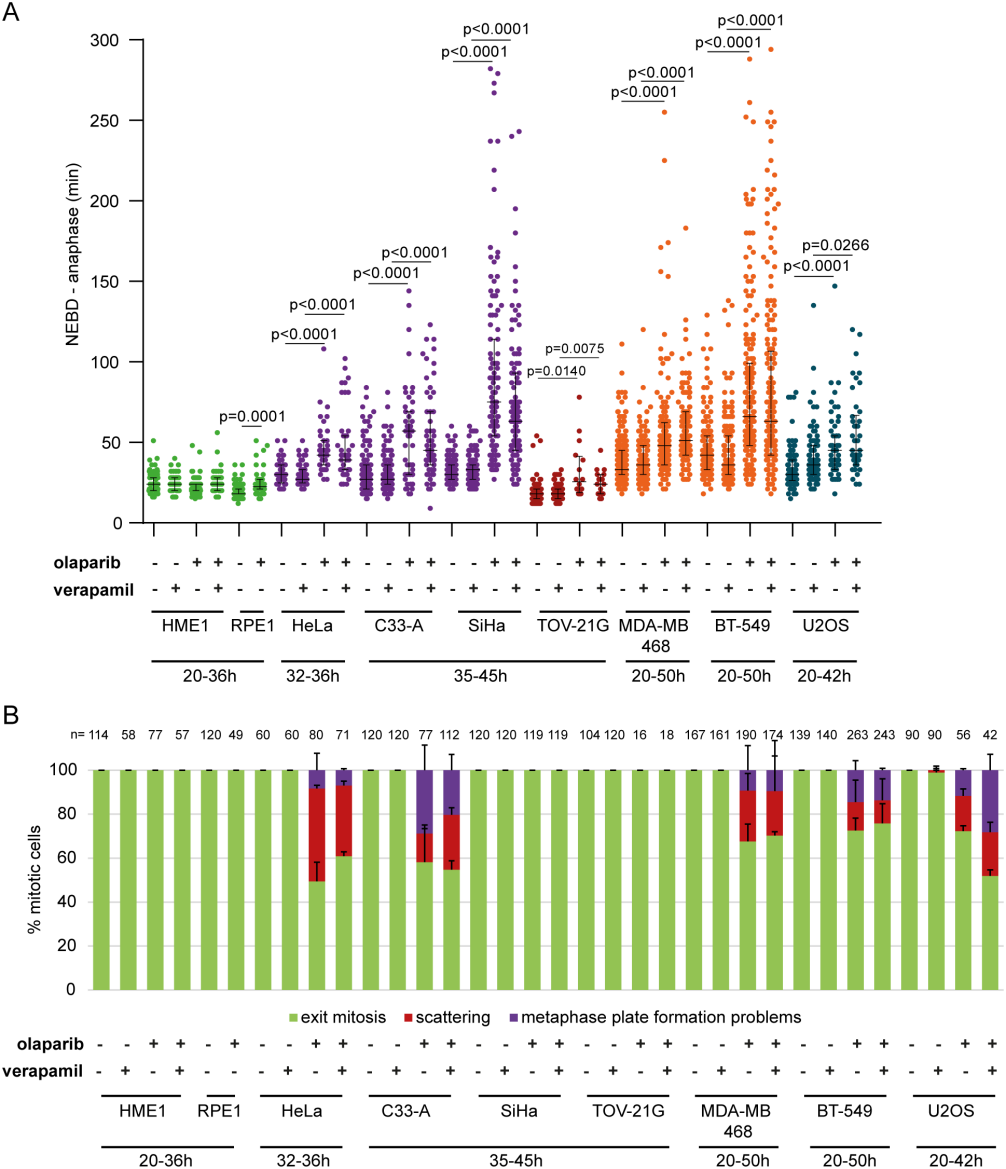
Olaparib causes anaphase delay and chromosome scattering in different cancer cell lines **(A)** Duration of NEBD-anaphase in non-cancerous and cancerous cell lines after verapamil, olaparib or concomitant verapamil and olaparib treatment. Live-cell imaging was always performed for 50 h after olaparib addition. Different time frames were analysed for different cell lines for the following reasons. Earlier time points were analysed in the case of HME1 and RPE1, which did not divide after 36 h. Later time points were analysed in cell lines that were highly stalled by olaparib treatment. Green = non-cancerous cell lines, violet = cervical cancer cell lines, dark red = ovarian cancer cell line, orange = breast cancer cell lines and blue = osteosarcoma cancer cell line **(B)** Percentage of the observed mitotic phenotypes.

Given that inhibition of drug efflux channels by verapamil was previously shown to decrease the survival of MRN-deficient colon cancer cells (HCT116 cells) treated with a PARP inhibitor [52], we also tested whether verapamil would potentiate the mitotic phenotypes caused by olaparib in different cell lines (Figure 6).

Olaparib reduced PARylation in all tested cell lines (Supplementary Figure 6). 10 μM olaparib did not induce scattering in HME1 GFP-H2B and RPE1 mRFP-H2B cells, while RPE1 cells exhibited a minor anaphase delay (7 min; Figure 6). Olaparib treatment in TOV-21G resulted in anaphase delay (45 min and 8 min, respectively) without causing chromatid scattering (Figure 6). Anaphase delay coupled with chromatid scattering was observed in C33-A, MDA-MB-468, BT549 and U2OS cells (Figure 6). Chromatid scattering was most pronounced in HeLa (32±2%), followed by C33-A (25±3%), MDA-MB-468 (20±16%), U2OS (20±5%), and BT-549 (11±10%) after concomitant treatment with olaparib and verapamil (Figure 6B).

Chromatid scattering does not correlate with the p53 status (Supplementary Table 2 and Supplementary Figure 7). Furthermore, expression levels of proteins involved in HR such as BRCA1, BRCA2, MRE11, RAD50 or NBS1 did not correlate with scattering either, although all cell lines that showed scattering exhibited low or no expression of at least one of these HR proteins (Supplementary Figure 7). This may suggest that inability to efficiently repair DNA damage arising through PARP trapping at replication forks leads to loss of cohesion at compromised DNA regions, followed by mitotic chromatid scattering.

To examine whether the degree of olaparib-induced chromatid scattering correlates with its cytotoxicity, we determined survival of all cell lines after exposure to a range of olaparib concentrations using colony formation assay and MTS assay to determine 14-day and 3-day cytotoxicity, respectively (Supplementary Figure 8). The degree of scattering correlated with cytotoxicity and HeLa was most sensitive to olaparib among cell lines that showed scattering (Supplementary Figure 8). However, cell lines that did not show scattering or other mitotic phenotypes, such as RPE1 and TOV-21G, were more sensitive to olaparib than HeLa, which suggests that in these cell lines olaparib induces cell death via non-mitotic pathways (Supplementary Figure 8). This is not surprising given that different agents can cause cell death via different pathways (e.g., apoptosis, necrosis, mitotic catastrophe, autophagy) in different cell types depending on their genotypic and phenotypic properties [53, 54].

### Chromatid scattering correlates with high PARP1 and PARP2 expression levels

**Figure 7:**
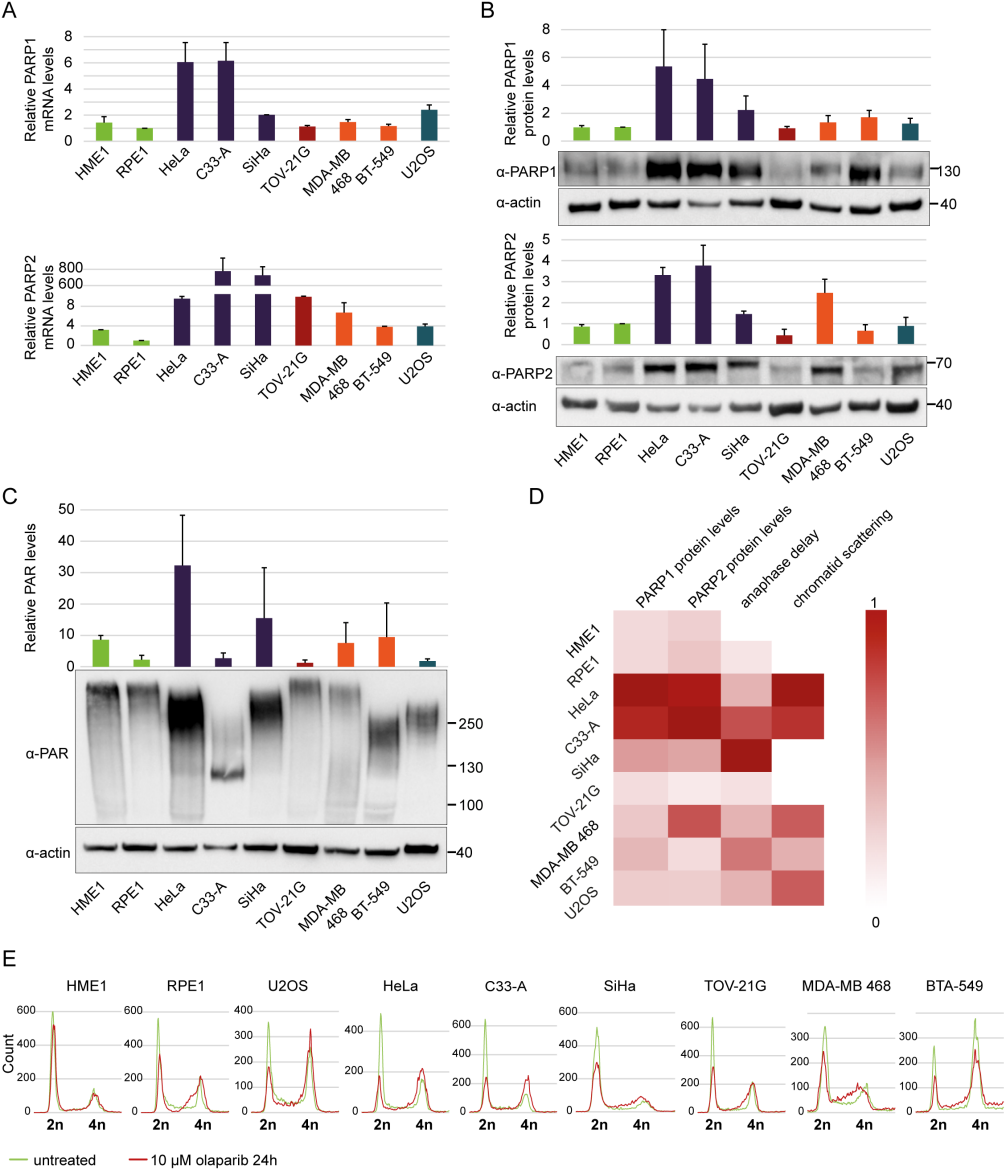
Cervical cancer cell lines HeLa and C33-A have high PARP1 and PARP2 mRNA and protein levels and are arrested in G2/M phase after olaparib treatment **(A)** Relative PARP1 and PARP2 mRNA levels normalized to actin as a loading control. **(B)** Western blot analysis of PARP1 and PARP2 protein levels normalized to actin as a loading control. **(C)** Relative PAR levels normalized to actin as a loading control. The quantification is based on blots showing total PAR generated not only by PARP1/2. **(D)** Heat map showing correlations between scattering caused by olaparib and relative PARP1 and PARP2 protein levels. All values were normalized from 0-1. Dark red (1) represents the highest protein levels, strongest anaphase delay and highest degree of scattering, while white (0) represents the lowest values. **(E)** Flow cytometry analysis of cancerous and non-cancerous cell lines after olaparib treatment.

The degree of cytotoxicity of PARP1 inhibitors was previously found to correlate with PARP1 expression levels, which are known to be increased in breast cancer [55]. Reduced PARP1 expression levels were associated with resistance to PARP inhibitors [56]. To test whether the scattering phenotype correlates with PARP expression levels, we quantified PARP1 and PARP2 mRNA levels in different cell lines by RT-qPCR and PARP1 and PARP2 protein levels by Western blotting (Figure 7A, B). Both mRNA and protein levels were very high in cell lines of cervical origin, particularly HeLa and C33-A, which also exhibited the highest degree of chromatid scattering (Figure 7A, B). PAR levels were highest in HeLa but low in C33-A, indicating that high PARP protein levels do not imply high catalytic activity (Figure 7C). Cell cycle analysis by flow cytometry revealed olaparib-induced S-phase stalling and G2/M arrest in all tested cell lines with the exception of HME1 (Figure 7E). Collectively, these data show that olaparib-induced chromatid scattering during mitosis correlates with high PARP1 and PARP2 expression levels (Figure 7D).

### Chromatid scattering does not correlate with the degree of DNA damage caused by olaparib

**Figure 8:**
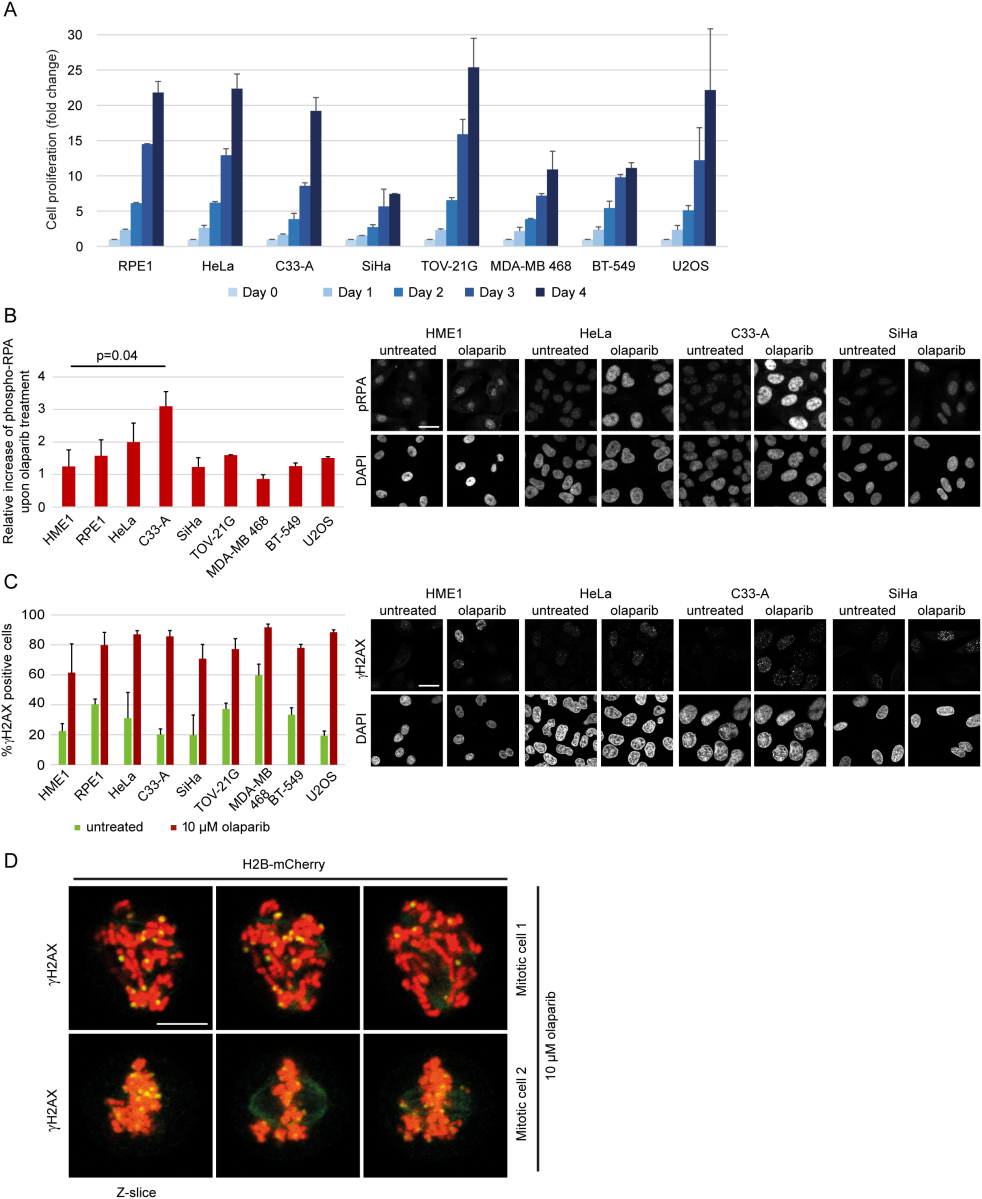
Replication stress and DNA damage after olaparib treatment **(A)** Growth rate of various cell lines determined by cell counting over 4 days. **(B)** Relative increase of phospho-RPA levels in untreated vs 10 μM olaparib-treated cells for 30 h. Immunofluorescent phospho-RPA intensities were measured by ImageJ (n>100). Representative images for HME1 and cervical cell lines are shown. **(C)** Percentage of γH2AX positive cells in untreated and 10 μM olaparib-treated cells for 30 h. Cells were scored as γH2AX positive if the number of foci per nucleus was ≥5 (n>100). Representative images for HME1 and cervical cell lines are shown. **(D)** Representative images of γH2AX foci in mitotic cells treated with 10 μM olaparib for 30 h. Scale bar=10 μm.

Given that cancer cells may have increased proliferation and increased levels of replication stress, which makes them more sensitive to ATR or Chk1 inhibitors [57, 58], we tested whether the propensity of different cell lines towards chromatid scattering correlates with elevated growth rate and elevated replication stress upon olaparib treatment. The growth rate was determined by counting the number of cells over four days (Figure 8A). We did not observe a correlation between the proliferation rate and the degree of scattering upon olaparib treatment among different cell lines (Figure 8A). Although we did not find a general correlation between growth rate and scattering, SiHa is an exceptionally slow-growing cell line, which could explain why it does not exhibit the scattering phenotype despite high PARP levels. To determine the levels of replication stress, we measured the total levels of phosphorylated RPA (replication protein A), which is found at single-stranded DNA generated at replication forks upon uncoupling of DNA helicase from DNA polymerase [59]. Phospho-RPA levels were two-fold increased in olaparib-treated HeLa and C33-A, which also showed the highest degree of chromatid scattering (Figure 8B and Figure 6). Other cell lines did not show a correlation between phospho-RPA and scattering (Figure 8B). We also compared the number of γH2AX foci-positive cells in untreated versus olaparib-treated cells and observed a similar increase in all cell lines (Figure 8C), as previously reported for olaparib-treated malignant lymphocyte cell lines [40]. γH2AX foci were also found in olaparib-treated mitotic cells that exhibited chromatid scattering (Figure 8D). However, the levels of γH2AX foci did not correlate with the degree of scattering upon olaparib treatment (Figure 8C). Differences in sensitivity to olaparib-induced chromatid scattering can thus be partially linked with the induction of replication stress but not the proliferation rate nor the levels of DNA damage. Replication stress was recently also detected upon olaparib treatment of MYCN-amplified neuroblastoma, which also express high PARP1/2 levels [60]. This provides further support for the model whereby high PARP1 and PARP2 levels are conducive to PARP entrapment and replication blockage resulting in cohesion weakening and mitotic chromatid scattering after olaparib treatment.

### Sister chromatid scattering is a common outcome of replication fork perturbation

**Figure 9:**
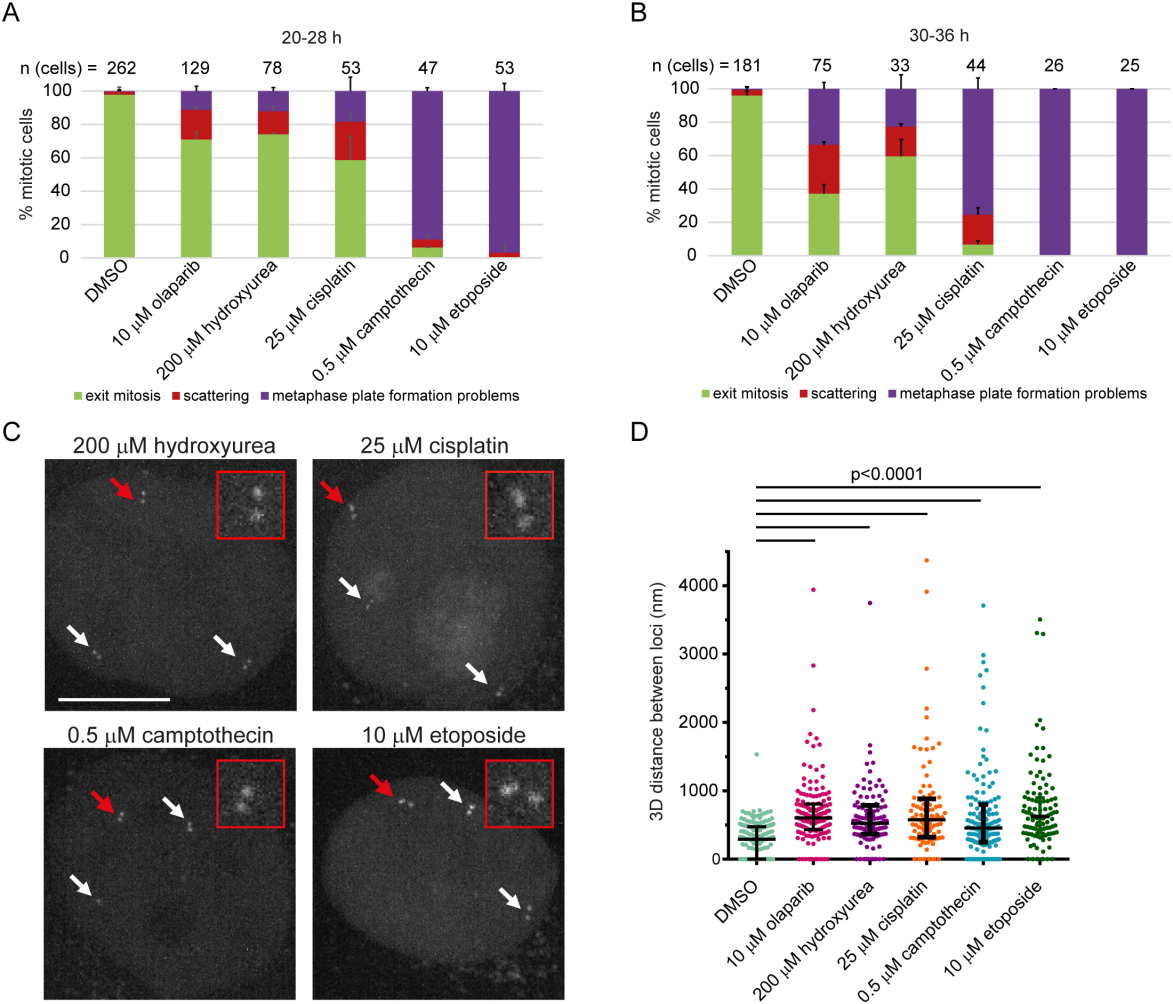
Sister chromatid scattering is a general outcome of replication fork perturbations induced by various agents in HeLa **(A, B)** Percentage of different mitotic phenotypes observed after (A) 20-28 h or (B) 30-36 h treatment with replication stress-inducing agents. **(C)** 3D distance between sister chromatids at Muc4 loci measured after live-cell imaging of HeLa cells treated with the same agents. Experimental workflow was as in Figure 5B for all agents except for hydroxyurea: 12 h after mitotic shake-off when cells are in S-phase agents were added for 4 h before imaging G2 cells. Cells were treated with 200 μM hydroxyurea for 20 h followed by 6 h recovery. **(D)** Representative images quantified under (C)**.**

In order to assess whether chromatid scattering induced by PARP1/2 inhibitors is a common consequence of replication stress-inducing agents, we tested the effect of hydroxyurea, cisplatin, campthotecin and etoposide on cell division. Hydroxyurea induces replication fork stalling by inhibiting ribonucleotide reductase and thereby depleting dNTP pools [61]. Cisplatin generates DNA crosslinks, while camptothecin and etoposide inhibit topoisomerase I and II thereby inducing single-strand and double-strand DNA breaks, respectively [62, 63]. Unlike hydroxyurea, cisplatin, camptothecin and etoposide directly damage DNA, which causes replication fork stalling and collapse in dividing cells [62–64]. To test the effect of these agents on mitotic progression by live imaging, we used the lowest concentration that induced pronounced S-phase stalling according to flow cytometry (Supplementary Figure 9). All agents induced chromatid scattering and metaphase plate formation problems, albeit to a different extent; hydroxyurea resulted in comparable levels of chromatid scattering and metaphase plate formation problems, whereas metaphase plate formation problems was the predominant mitotic phenotype of cisplatin, camptothecin and etoposide that directly damage DNA (Figure 9A, B). Longer treatments showed an increase in metaphase plate formation problems, presumably due to an increase in DNA damage (Figure 9A, B). This suggests that chromatid scattering is a common outcome of replication stress-inducing agents, whereas metaphase plate formation problems arises as a dominant phenotype of agents that directly damage DNA. All agents weakened sister chromatid cohesion as revealed by the measurement of sister chromatid distances in G2 cells (Figure 9C, D). The median distance between separated sister loci increased from 292 nm (n=163) in untreated cells to 526 nm (n=111) in hydroxyurea-treated cells, 578 nm (n=102) in cisplatin-treated cells, 454 nm (n=142) in camptothecin-treated cells and 621 nm (n=105) in etoposide-treated cells (Figure 9D). Taken together, olaparib and replication stress-inducing agents weaken sister chromatid cohesion in interphase, resulting in premature loss of cohesion in mitosis.

## DISCUSSION

**Figure 10:**
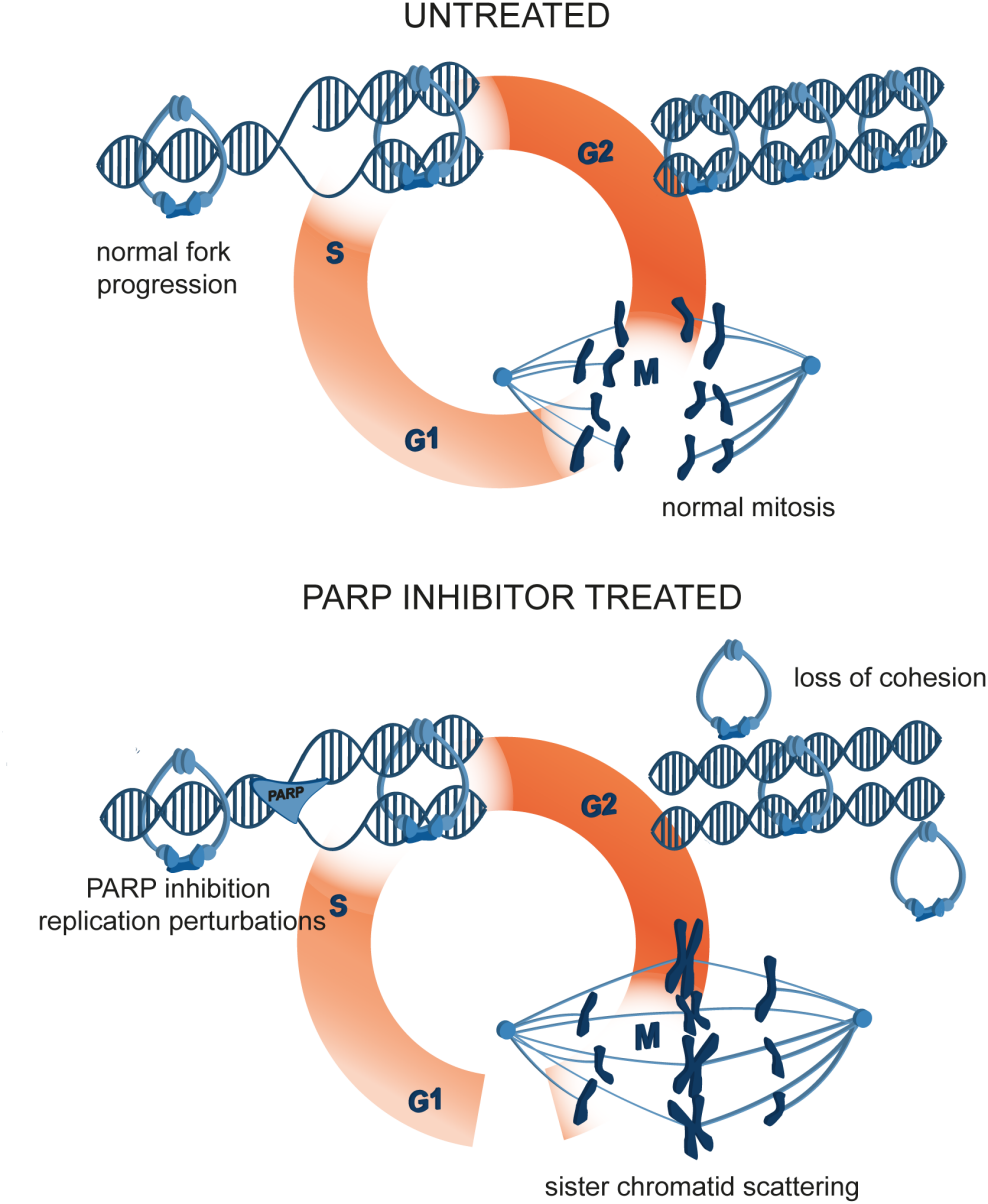
A model of mitotic cell death caused by premature loss of cohesion due to PARP inhibition with olaparib PARP inhibition causes replication fork stalling and premature loss of cohesion in interphase. Mitotic cells are consequently arrested in metaphase, sister chromatids scatter away from the metaphase plate and the cells eventually die.

Numerous clinical trials have been conducted since the initial development of PARP inhibitors [65], which culminated in the approval of olaparib for the treatment of BRCA-mutated ovarian cancer [66]. However, the molecular basis of PARP inhibitor function remains unclear [67, 68]. PARP1, PARP2, PARP3 and PARP5a (tankyrase) co-localization with various mitotic structures prompted us to study the effect of their inhibition or depletion on mitosis. By tracking mitotic progression of individual cells, we uncovered a new phenotype of PARP1/2 inhibition by olaparib. We showed that, by acting on replicating cells, olaparib induces metaphase arrest and sister chromatid scattering, ultimately resulting in cell death (Figure 10). Moreover, we delineated the mechanism of mitotic scattering by showing that olaparib causes loss of sister chromatid cohesion in G2 cells. The olaparib-induced scattering phenotype is suppressed by PARP1 or PARP2 depletion, demonstrating that PARP1 and PARP2 are the relevant targets and that PARP1/2 trapping rather than catalytic inhibition is the mechanism of olaparib-induced mitotic failure. Chromatid scattering was observed in various cancer cell lines and was correlated with PARP1 and PARP2 expression levels.

The PARP inhibitor olaparib is the first clinically approved drug that targets DNA damage response (DDR) [66]. Olaparib is particularly efficient in tumors lacking specific DDR functions, which renders them more reliant on a particular DDR pathway. In particular, patients bearing mutations in BRCA1/BRCA2 genes that are required for the homologous recombination DNA repair pathway have shown exceptional susceptibility to PARP inhibitors. Multiple clinical trials carried out since 2009 have demonstrated the beneficial effects of olaparib on BRCA-mutated ovarian and breast cancer, as well as prostate and pancreatic cancer, Ewing’s sarcoma, small cell lung carcinoma and neuroblastoma [18, 69]. Given that cancers without mutations in DNA repair pathways are also susceptible to PARP inhibition, non-DNA repair functions of PARP1/2 are likely also responsible for the deleterious effects of PARP inhibition [18]. Indeed, our study shows that olaparib induces chromatid scattering in metaphase-arrested cells resulting in cell death. However, the observed olaparib-induced mitotic phenotype is not caused by PARP1/2 inhibition in mitosis but instead results from PARP1/2 inhibition during S-phase replication.

Contrary to olaparib treatment, PARP1 or PARP2 depletion did not result in chromatid scattering. Differential effects of PARP inhibition and PARP depletion have been attributed to the entrapment of PARP1 on DNA by inhibitors such as olaparib and talazoparib [13]. By interacting with the D-loop at the outer border of the NAD site, olaparib and talazoparib induce conformational changes in the PARP1 DNA-binding domains to stabilize the PARP1-DNA complex [70]. Trapped PARP-DNA complexes prevent DNA replication and transcription; PARP poisoning effect therefore determines the potency of PARP inhibitors rather than their effect on PARP catalytic inhibition [13, 67]. As chromatid scattering results from olaparib-induced loss of cohesion in S/G2 phase that can be rescued by PARP1/2 depletion, we surmise that olaparib-induced chromosome scattering is caused by PARP1/2 trapping. This is further supported by a correlation between high PARP1 and PARP2 levels, induction of replication stress and chromatid scattering in HeLa and C33-A cell lines.

Replication problems have been already linked with mitotic defects [71]. Faithful segregation of sister chromatids can be compromised by incompletely replicated chromosomes caused by replication fork stalling, incompletely resolved DNA repair intermediates or topologically intertwined sister chromatids [71]. Mitotic structures caused by replication problems include anaphase chromatin bridges and ultrafine DNA bridges [71]. So far, metaphase arrest and premature loss of cohesion have not been linked with replication problems. By extending our analyses to other agents that perturb replication fork progression, such as hydroxyurea, cisplatin and topoisomerase inhibitors, we showed for the first time that replication problems can also lead to premature loss of cohesion in metaphase-arrested cells.

Genetic predisposition (e.g., BRCA mutations) or phenotypic characteristics (e.g., platinum resistance) are not sufficient to predict patient response to olaparib treatment [58]. Chromatid scattering was observed in cervical cancer cells with increased PARP1 and PARP2 protein levels (HeLa, C33-A), suggesting that PARP1 and PARP2 protein levels could be used as a predictive biomarker for the efficiency of olaparib treatment, as previously proposed [60, 67]. Olaparib is currently undergoing various clinical trials, including different gynaecological malignancies such as cervical and uterine cancer [72], where such a biomarker may be particularly useful. However, one of the three tested cervical cancer cell lines, SiHa, did not exhibit chromatid scattering despite high PARP levels, which is most likely due to its slow proliferation and low susceptibility to replication stress as a result. A larger panel of cell lines with variable PARP levels and proliferation rates would need to be analysed to draw a definite conclusion.

In summary, we showed that sister chromatid scattering in mitosis is a new mechanism of olaparib-induced cytotoxicity. By entrapping PARP1 on replicating DNA, olaparib obstructs replication fork progression resulting in loss of sister chromatid cohesion in G2 cells (Figure 10). Loss of cohesion in interphase cells causes chromatid scattering in metaphase cells, metaphase arrest and cell death.

## MATERIALS AND METHODS

### RNAi and inhibitors

Cells were seeded and transfected 24 h prior to live imaging in the presence of different siRNAs (30-50 nM) using RNAiMAX (Invitrogen). EGFP-PCNA transfection was performed 24 h prior to imaging using FugeneHD (Promega). The following inhibitors were used: olaparib (AZD2281, Ku-0059436; Selleckchem), veliparib (ABT-888; Selleckchem), talazoparib (BMN 673; Selleckchem); XAV-939 (Selleckchem), ME328 [34], verapamil (Sigma), MG132 (Sigma), hydroxyurea (Sigma), cisplatin (CPDD, Sigma), (S)-(+)-camptothecin (Sigma), etoposide (Sigma). SiR-Hoechst (Life Technologies) was used at the final concentration of 500 nM.

### Generation of EGFP-PARP1 and EGFP-PARP2 overexpressing cell lines using lentiviral system

EGFP-PARP1 and EGFP-PARP2 were cloned into a transfer plasmid under the EF1a promoter. HEK 293NT cells were transfected with transfer plasmid, viral envelope coding plasmid and lentiviral packaging plasmid using PEI. 36 h after transfection virus-containing supernatant was filtered and added to HeLa RIEP cells (Day 1). Second infection was performed 24 h later (Day 2). On Day 3 cells were washed with PBS and placed in fresh media. On Day 6 washing was repeated and GFP-positive cells were FACS sorted.

### Microscopy

Live-cell wide-field microscopy experiments on Molecular Devices ImageXpress Micro screening microscope were performed using 96-well plates as already described [51]. Live-cell spinning disc microscopy was performed using 4-well glass bottom dishes (Greiner) in an environmentally controlled chamber with an Axio Observer Z1 (Zeiss) inverted microscope equipped with an EM-CCD camera (Evolve EM-512), Yokogawa CSU-X1-A1 spinning disc unit (pinhole diameter 50 μm), 488 nm diode laser, 561 nm DPSS laser (AOTF-controlled) and a Plan-Apochromat 63x/1.4 oil-immersion objective. Cells were maintained at 5% CO_2_ and 37°C during experiments. Confocal microscopy was performed on a customized Zeiss LSM 710 microscope using an x63, 1.4N.A oil Plan-Apochromat objective (Zeiss).

### Immunofluorescence

Cells were grown on high-precision borosilicate cover glasses (LH22.1 Roth Labware). For cyclin B staining (1:200; Cell Signalling), cells were fixed in PTEMF buffer or in 4% PFA followed by ice-cold methanol, and stained as previously described [29]. For γH2AX and phospho-RPA staining, cells were fixed in 4% PFA, permeabilised in 0.5% Triton X-100 in PBS and blocked for one hour in 0.1% Tween and 1% BSA, followed by incubation with primary antibodies: rabbit anti-γH2AX (1:500; Bethyl) or rabbit anti-phospho-RPA S4/S8 (1:500; Bethyl) and subsequent incubation with appropriate secondary antibodies (1:500; Alexa Fluor^®^).

### Image and statistical analysis

Image analysis was performed using FiJi (ImageJ v1.5). Duration from NEBD (nuclear envelope breakdown) to anaphase (min) is presented using median with interquartile range. In Figure 5A box and whiskers are plotted and presented as 10-90 percentile. Statistical analysis was performed using Kolmogorov-Smirnov test. Live imaging microscopy data are based on at least two biological replicates with three or more technical replicates each.

### FACS

Cell cycle profiling was performed using propidium iodide staining as described [46], measured at Zytofluorometer FACSCalibur and analysed using Flowing Software 2.5.1.

### Sister chromatid distance measurements

5x10^5^ Muc4 TRE3G-dCas9-mEGFP cells [45] were seeded in T75 flasks and dCas9-mEGFP expression induced with 1 μg/mL doxycycline for 48 h. Shake-off and collection of mitotic cells was performed as follows. After a pre-shake-off and 2x wash with pre-warmed PBS, the cells were allowed to enter mitosis for 2 h in pre-warmed medium with 1 μg/mL doxycycline. Mitotic cells were mechanically detached by shaking the plates, centrifuged at 500 g for 5 min, resuspended in an appropriate amount of medium containing 1 μg/mL doxycycline and seeded in 4-chamber glass bottom dishes (Greiner). For silencing experiments siRNA and RNAiMAX reagent were mixed and incubated as described above and added to the glass bottom dish prior to seeding mitotic cells. Live imaging was performed 16 h after the mitotic shake-off when the cells entered G2 phase. 10 μM olaparib was added 4 h prior to imaging when the cells were in S-phase (12 h after mitotic shake-off). Imaging was performed on a spinning disc microscope with an sCMOS 2xpco.edge 4.2 camera (0.065 μm pixel size) and an EC Plan-Neofluar 100x/1.3 oil-immersion objective. Z-stack images were acquired in 100 nm intervals. The 3D distance of paired sister chromatids was determined with the Fiji Plugin Trackmate (DoG detector, sub-pixel localization, estimated spot diameter of 150 nm) by using the x, y and z coordinates of the detected sister chromatids to calculate the distance d=sqrt( (X_1_-X_2_)^2^ + (Y_1_-Y_2_)^2^ + (Z_1_-Z_2_)^2^).

### Chromosome spreads

Chromosome spreading and Giemsa staining was performed as previously described [46, 73], excluding the nocodazole treatment. In total over 6600 prometaphase/metaphase spreads were analyzed in two independent experiments (each containing five technical replicates) by automated analysis using Metafer4 v 2.12.116 (MetaSystems). All slides were scanned automatically and spreads were randomly selected in each technical replicate using Metafer4. Spreads were then blindly categorized into four phenotypes.

### Quantitative RT-PCR

Total RNA was extracted with TRI reagent (Sigma) and PCl (Phenol equilibrated, stabilized: Chloroform: Isoamyl alcohol 25:24:1; AppliChem), and precipitated in ethanol. RT (reverse-transcription) reaction was performed with random primers (Invitrogen 48190_011) and 5xProtoScript II RT (BioLabs). Quantitative PCR was performed with 5xHot FirePol Eva Green qPCR mix (BioZyme). All experiments were repeated twice with two technical replicates.

### Immunoblotting

Cell pellets were lysed in 50 mM Tris pH8, 150 mM NaCl, 1% Triton, 1 mM DTT, 50 units/ml benzonase (Novagen) and protease inhibitors (EDTA-free, Roche). Mouse anti-tubulin (1:5000; Sigma), mouse anti-actin (1:20000; Sigma), rabbit anti-PARP1 (1:1000; Cell Signalling), rabbit anti-PAR (1:1000; Trevigen), mouse anti-PARP2 (1:50; Enzo), rabbit anti-PARP3 (1:200; Dantzer lab), rabbit tankyrase-1/2 H-350 (1:200; Santa Cruz), rabbit anti-Cdc20 (1:1000; Peters lab), rabbit anti-human sororin (1:1000; Peters lab), rabbit anti-SMC3 (1:1000; Bethyl laboratories), mouse anti-acetyl-SMC3 (1:1000; Shirahige lab), rabbit anti-P53 (1:1000; Cell Signaling), rabbit anti-BRCA1 (1:1000; Cell Signaling), rabbit anti-BRCA2 (1:1000; Cell Signaling), rabbit anti-RAD50 (1:1000; Cell Signaling), rabbit anti-MRE11 (1:1000; Cell Signaling), rabbit anti-NBS1 (1:1000; Cell Signaling). Secondary HRP-conjugated antibodies (Jackson ImmunoResearch) or IRDye fluorescent dye antibodies (LI-COR) were used at 1:10000 dilution. Images were taken on ChemiDoc Touch Imaging System (Bio-Rad) or Odyssey CLx imager (LI-COR) using Image Lab 5.2.1 for analysis. Quantification of Western blots is based on at least three independent experiments. Error bars represent standard deviation.

### Colony formation assay

Cells were seeded and treated with different concentrations of olaparib at a very low confluence (1000-3000 cells per 60 mm dish). Medium (with or without olaparib) was exchanged every 4-5 days. After 14 days, medium was removed and cells were fixed with 4% PFA and incubated 10 min RT. PFA was removed and 0.1% crystal violet in 25% methanol was added to the dish and incubated 20 min at 4ºC. Crystal violet was removed and dishes were washed in filtered water until residual crystal violet was completely removed. Percentage of surviving cells was quantified by measuring the area of colonies using the ImageJ-plugin ‘ColonyArea’. The intensity of colonies was not taken into account due to variable staining of different cell lines by crystal violet. The quantification is based on at least two independent experiments with two technical replicates each.

### MTS assay

HeLa cells were seeded in 96-well plates (1000 cells per well) in the presence of siRNAs. Olaparib was added 24 h later. For the comparison of survival across cell lines, RPE1, HME1, HeLa, TOV-21G, BT-549 and U2OS cells were seeded at 1000 cells per well; SiHa was seeded at 2000 cells per well; MDA-MB 468 was seeded at 4000 cells per well; C33-A was not measured as it does not metabolize the MTS reagent. Olaparib was added during seeding. CellTiter 96^®^solution (Promega) was added after 72 h and the absorbance was measured at 490 nm. The quantification is based on three independent experiments with three technical replicates each.

### Proliferation assay

5x10^4^ cells were seeded in one well of a 6-well plate on day zero. After 24 h cells were trypsinized, resuspended in DMEM media and counted using Mini Automated Cell Counter (ORFLO). Counting was repeated each 24 h for 4 consecutive days. Cell proliferation was quantified by normalizing the number of cells with the number of cells on day zero.

## SUPPLEMENTARY MATERIALS FIGURES AND TABLES







## References

[1] De Vos M, Schreiber V, Dantzer F (2012). The diverse roles and clinical relevance of PARPs in DNA damage repair: current state of the art. Biochem Pharmacol.

[2] Luo X, Kraus WL (2012). On PAR with PARP: cellular stress signaling through poly(ADP-ribose) and PARP-1. Genes Dev.

[3] Kraus WL, Hottiger MO (2013). PARP-1 and gene regulation: Progress and puzzles. Mol Aspects Med.

[4] Barkauskaite E, Jankevicius G, Ahel I (2015). Structures and Mechanisms of Enzymes Employed in the Synthesis and Degradation of PARP-Dependent Protein ADP-Ribosylation. Mol Cell.

[5] Gibson BA, Kraus WL (2012). New insights into the molecular and cellular functions of poly(ADP-ribose) and PARPs. Nature.

[6] Ame JC, Rolli V, Schreiber V, Niedergang C, Apiou F, Decker P, Muller S, Hoger T, Menissier-de Murcia J, de Murcia G (1999). PARP-2, A novel mammalian DNA damage-dependent poly(ADP-ribose) polymerase. J Biol Chem.

[7] Beck C, Robert I, Reina-San-Martin B, Schreiber V, Dantzer F (2014). Poly(ADP-ribose) polymerases in double-strand break repair: focus on PARP1, PARP2 and PARP3. Exp Cell Res.

[8] Caldecott KW (2014). Protein ADP-ribosylation and the cellular response to DNA strand breaks. DNA Repair.

[9] Bryant HE, Petermann E, Schultz N, Jemth AS, Loseva O, Issaeva N, Johansson F, Fernandez S, McGlynn P, Helleday T (2009). PARP is activated at stalled forks to mediate Mre11-dependent replication restart and recombination. Embo J.

[10] Yang YG, Cortes U, Patnaik S, Jasin M, Wang ZQ (2004). Ablation of PARP-1 does not interfere with the repair of DNA double-strand breaks, but compromises the reactivation of stalled replication forks. Oncogene.

[11] Berti M, Ray Chaudhuri A, Thangavel S, Gomathinayagam S, Kenig S, Vujanovic M, Odreman F, Glatter T, Graziano S, Mendoza-Maldonado R, Marino F, Lucic B, Biasin V (2013). Human RECQ1 promotes restart of replication forks reversed by DNA topoisomerase I inhibition. Nature.

[12] Ray Chaudhuri A, Hashimoto Y, Herrador R, Neelsen KJ, Fachinetti D, Bermejo R, Cocito A, Costanzo V, Lopes M (2012). Topoisomerase I poisoning results in PARP-mediated replication fork reversal. Nature.

[13] Murai J, Huang SY, Das BB, Renaud A, Zhang Y, Doroshow JH, Ji J, Takeda S, Pommier Y (2012). Trapping of PARP1 and PARP2 by Clinical PARP Inhibitors. Cancer Res.

[14] Ashworth A (2008). A synthetic lethal therapeutic approach: poly(ADP) ribose polymerase inhibitors for the treatment of cancers deficient in DNA double-strand break repair. J Clin Oncol.

[15] Farmer H, McCabe N, Lord CJ, Tutt AN, Johnson DA, Richardson TB, Santarosa M, Dillon KJ, Hickson I, Knights C, Martin NM, Jackson SP, Smith GC, Ashworth A (2005). Targeting the DNA repair defect in BRCA mutant cells as a therapeutic strategy. Nature.

[16] Bryant HE, Schultz N, Thomas HD, Parker KM, Flower D, Lopez E, Kyle S, Meuth M, Curtin NJ, Helleday T (2005). Specific killing of BRCA2-deficient tumours with inhibitors of poly(ADP-ribose) polymerase. Nature.

[17] O'Neil NJ, van Pel DM, Hieter P (2013). Synthetic lethality and cancer: cohesin and PARP at the replication fork. Trends Genet.

[18] Weaver AN, Yang ES (2013). Beyond DNA Repair: Additional Functions of PARP-1 in Cancer. Front Oncol.

[19] Saxena A, Wong LH, Kalitsis P, Earle E, Shaffer LG, Choo KH (2002). Poly(ADP-ribose) polymerase 2 localizes to mammalian active centromeres and interacts with PARP-1, Cenpa, Cenpb and Bub3, but not Cenpc. Hum Mol Genet.

[20] Halappanavar SS, Shah GM (2004). Defective control of mitotic and post-mitotic checkpoints in poly(ADP-ribose) polymerase-1(-/-) fibroblasts after mitotic spindle disruption. Cell Cycle.

[21] Kanai M, Tong WM, Sugihara E, Wang ZQ, Fukasawa K, Miwa M (2003). Involvement of poly(ADP-Ribose) polymerase 1 and poly(ADP-Ribosyl)ation in regulation of centrosome function. Mol Cell Biol.

[22] Simbulan-Rosenthal CM, Haddad BR, Rosenthal DS, Weaver Z, Coleman A, Luo R, Young HM, Wang ZQ, Ried T, Smulson ME (1999). Chromosomal aberrations in PARP(-/-) mice: genome stabilization in immortalized cells by reintroduction of poly(ADP-ribose) polymerase cDNA. Proc Nat Acad Sci USA.

[23] Yang F, Baumann C, De La Fuente R (2009). Persistence of histone H2AX phosphorylation after meiotic chromosome synapsis and abnormal centromere cohesion in poly (ADP-ribose) polymerase (Parp-1) null oocytes. Dev Biol.

[24] Kashima L, Idogawa M, Mita H, Shitashige M, Yamada T, Ogi K, Suzuki H, Toyota M, Ariga H, Sasaki Y, Tokino T (2012). CHFR protein regulates mitotic checkpoint by targeting PARP-1 protein for ubiquitination and degradation. J Biol Chem.

[25] Cook BD, Dynek JN, Chang W, Shostak G, Smith S (2002). Role for the related poly(ADP-Ribose) polymerases tankyrase 1 and 2 at human telomeres. Mol Cell Biol.

[26] Chang P, Coughlin M, Mitchison TJ (2005). Tankyrase-1 polymerization of poly(ADP-ribose) is required for spindle structure and function. Nat Cell Biol.

[27] Boehler C, Gauthier LR, Mortusewicz O, Biard DS, Saliou JM, Bresson A, Sanglier-Cianferani S, Smith S, Schreiber V, Boussin F, Dantzer F (2011). Poly(ADP-ribose) polymerase 3 (PARP3), a newcomer in cellular response to DNA damage and mitotic progression. Proc Nat Acad Sci USA.

[28] Murai J, Huang SY, Renaud A, Zhang Y, Ji J, Takeda S, Morris J, Teicher B, Doroshow JH, Pommier Y (2014). Stereospecific PARP trapping by BMN 673 and comparison with olaparib and rucaparib. Mol Cancer Ther.

[29] Dick AE, Gerlich DW (2013). Kinetic framework of spindle assembly checkpoint signalling. Nat Cell Biol.

[30] Menear KA, Adcock C, Boulter R, Cockcroft XL, Copsey L, Cranston A, Dillon KJ, Drzewiecki J, Garman S, Gomez S, Javaid H, Kerrigan F, Knights C (2008). 4-[3-(4-cyclopropanecarbonylpiperazine-1-carbonyl)-4-fluorobenzyl]-2H-phthalazin- 1-one: a novel bioavailable inhibitor of poly(ADP-ribose) polymerase-1. J Med Chem.

[31] Shen Y, Rehman FL, Feng Y, Boshuizen J, Bajrami I, Elliott R, Wang B, Lord CJ, Post LE, Ashworth A (2013). BMN 673, a novel and highly potent PARP1/2 inhibitor for the treatment of human cancers with DNA repair deficiency. Clin Cancer Res.

[32] Donawho CK, Luo Y, Luo Y, Penning TD, Bauch JL, Bouska JJ, Bontcheva-Diaz VD, Cox BF, DeWeese TL, Dillehay LE, Ferguson DC, Ghoreishi-Haack NS, Grimm DR (2007). ABT-888, an orally active poly(ADP-ribose) polymerase inhibitor that potentiates DNA-damaging agents in preclinical tumor models. Clin Cancer Res.

[33] Huang SM, Mishina YM, Liu S, Cheung A, Stegmeier F, Michaud GA, Charlat O, Wiellette E, Zhang Y, Wiessner S, Hild M, Shi X, Wilson CJ (2009). Tankyrase inhibition stabilizes axin and antagonizes Wnt signalling. Nature.

[34] Lindgren AE, Karlberg T, Thorsell AG, Hesse M, Spjut S, Ekblad T, Andersson CD, Pinto AF, Weigelt J, Hottiger MO, Linusson A, Elofsson M, Schuler H (2013). PARP inhibitor with selectivity toward ADP-ribosyltransferase ARTD3/PARP3. ACS Chem Biol.

[35] Daum JR, Potapova TA, Sivakumar S, Daniel JJ, Flynn JN, Rankin S, Gorbsky GJ (2011). Cohesion fatigue induces chromatid separation in cells delayed at metaphase. Curr Biol.

[36] Stevens D, Gassmann R, Oegema K, Desai A (2011). Uncoordinated loss of chromatid cohesion is a common outcome of extended metaphase arrest. PLoS One.

[37] Teixeira LK, Reed SI (2013). Ubiquitin ligases and cell cycle control. Annu Rev Biochem.

[38] Thorsell AG, Ekblad T, Karlberg T, Low M, Pinto AF, Tresaugues L, Moche M, Cohen MS, Schuler H (2017). Structural Basis for Potency and Promiscuity in Poly(ADP-ribose) Polymerase (PARP) and Tankyrase Inhibitors. J Med Chem.

[39] Fong PC, Boss DS, Yap TA, Tutt A, Wu P, Mergui-Roelvink M, Mortimer P, Swaisland H, Lau A, O'Connor MJ, Ashworth A, Carmichael J, Kaye SB (2009). Inhibition of poly(ADP-ribose) polymerase in tumors from BRCA mutation carriers. N Engl J Med.

[40] Dale Rein I, Solberg Landsverk K, Micci F, Patzke S, Stokke T (2015). Replication-induced DNA damage after PARP inhibition causes G2 delay, and cell line-dependent apoptosis, necrosis and multinucleation. Cell Cycle.

[41] Strom CE, Johansson F, Uhlen M, Szigyarto CA, Erixon K, Helleday T (2011). Poly (ADP-ribose) polymerase (PARP) is not involved in base excision repair but PARP inhibition traps a single-strand intermediate. Nucleic Acids Res.

[42] Godon C, Cordelieres FP, Biard D, Giocanti N, Megnin-Chanet F, Hall J, Favaudon V (2008). PARP inhibition versus PARP-1 silencing: different outcomes in terms of single-strand break repair and radiation susceptibility. Nucleic Acids Res.

[43] Nishiyama T, Ladurner R, Schmitz J, Kreidl E, Schleiffer A, Bhaskara V, Bando M, Shirahige K, Hyman AA, Mechtler K, Peters JM (2010). Sororin mediates sister chromatid cohesion by antagonizing Wapl. Cell.

[44] Zhang N, Pati D (2015). C-terminus of Sororin interacts with SA2 and regulates sister chromatid cohesion. Cell Cycle.

[45] Chen B, Gilbert LA, Cimini BA, Schnitzbauer J, Zhang W, Li GW, Park J, Blackburn EH, Weissman JS, Qi LS, Huang B (2013). Dynamic imaging of genomic loci in living human cells by an optimized CRISPR/Cas system. Cell.

[46] van der Lelij P, Stocsits RR, Ladurner R, Petzold G, Kreidl E, Koch B, Schmitz J, Neumann B, Ellenberg J, Peters JM (2014). SNW1 enables sister chromatid cohesion by mediating the splicing of sororin and APC2 pre-mRNAs. EMBO J.

[47] Sundaramoorthy S, Vazquez-Novelle MD, Lekomtsev S, Howell M, Petronczki M (2014). Functional genomics identifies a requirement of pre-mRNA splicing factors for sister chromatid cohesion. EMBO J.

[48] Bouchet BP, Bertholon J, Falette N, Audoynaud C, Lamblot C, Puisieux A, Galmarini CM (2007). Paclitaxel resistance in untransformed human mammary epithelial cells is associated with an aneuploidy-prone phenotype. Br J Cancer.

[49] Wang Y, Lee YM, Baitsch L, Huang A, Xiang Y, Tong H, Lako A, Von T, Choi C, Lim E, Min J, Li L, Stegmeier F (2014). MELK is an oncogenic kinase essential for mitotic progression in basal-like breast cancer cells. ELife.

[50] Barisic M, Sohm B, Mikolcevic P, Wandke C, Rauch V, Ringer T, Hess M, Bonn G, Geley S (2010). Spindly/CCDC99 is required for efficient chromosome congression and mitotic checkpoint regulation. Mol Biol Cell.

[51] Lukinavicius G, Blaukopf C, Pershagen E, Schena A, Reymond L, Derivery E, Gonzalez-Gaitan M, D'Este E, Hell SW, Wolfram Gerlich D, Johnsson K (2015). SiR-Hoechst is a far-red DNA stain for live-cell nanoscopy. Nat Commun.

[52] Oplustilova L, Wolanin K, Mistrik M, Korinkova G, Simkova D, Bouchal J, Lenobel R, Bartkova J, Lau A, O'Connor MJ, Lukas J, Bartek J (2012). Evaluation of candidate biomarkers to predict cancer cell sensitivity or resistance to PARP-1 inhibitor treatment. Cell Cycle.

[53] Ricci MS, Zong WX (2006). Chemotherapeutic approaches for targeting cell death pathways. Oncologist.

[54] Fulda S, Debatin KM (2006). Extrinsic versus intrinsic apoptosis pathways in anticancer chemotherapy. Oncogene.

[55] Bieche I, Pennaneach V, Driouch K, Vacher S, Zaremba T, Susini A, Lidereau R, Hall J (2013). Variations in the mRNA expression of poly(ADP-ribose) polymerases, poly(ADP-ribose) glycohydrolase and ADP-ribosylhydrolase 3 in breast tumors and impact on clinical outcome. Int J Cancer.

[56] Pettitt SJ, Rehman FL, Bajrami I, Brough R, Wallberg F, Kozarewa I, Fenwick K, Assiotis I, Chen L, Campbell J, Lord CJ, Ashworth A (2013). A genetic screen using the PiggyBac transposon in haploid cells identifies Parp1 as a mediator of olaparib toxicity. PLoS One.

[57] Macheret M, Halazonetis TD (2015). DNA replication stress as a hallmark of cancer. Annu Rev Pathol.

[58] Helleday T (2013). Putting poly (ADP-ribose) polymerase and other DNA repair inhibitors into clinical practice. Curr Opin Oncol.

[59] Zeman MK, Cimprich KA (2014). Causes and consequences of replication stress. Nature Cell Biol.

[60] Colicchia V, Petroni M, Guarguaglini G, Sardina F, Sahun-Roncero M, Carbonari M, Ricci B, Heil C, Capalbo C, Belardinilli F, Coppa A, Peruzzi G, Screpanti I (2017). PARP inhibitors enhance replication stress and cause mitotic catastrophe in MYCN-dependent neuroblastoma. Oncogene.

[61] Merrick CJ, Jackson D, Diffley JF (2004). Visualization of altered replication dynamics after DNA damage in human cells. J Biol Chem.

[62] Pommier Y (2006). Topoisomerase I inhibitors: camptothecins and beyond. Nat Rev Cancer.

[63] Deans AJ, West SC (2011). DNA interstrand crosslink repair and cancer. Nat Rev Cancer.

[64] Saintigny Y, Delacote F, Vares G, Petitot F, Lambert S, Averbeck D, Lopez BS (2001). Characterization of homologous recombination induced by replication inhibition in mammalian cells. EMBO J.

[65] Nduka N, Skidmore CJ, Shall S (1980). The enhancement of cytotoxicity of N-methyl-N-nitrosourea and of gamma-radiation by inhibitors of poly(ADP-ribose) polymerase. Eur J Biochem.

[66] O'Connor MJ (2015). Targeting the DNA Damage Response in Cancer. Mol Cell.

[67] Shen Y, Aoyagi-Scharber M, Wang B (2015). Trapping Poly(ADP-Ribose) Polymerase. J Pharmacol Exp Ther.

[68] Feng FY, de Bono JS, Rubin MA, Knudsen KE (2015). Chromatin to Clinic: The Molecular Rationale for PARP1 Inhibitor Function. Mol Cell.

[69] Sonnenblick A, de Azambuja E, Azim HA, Piccart M (2015). An update on PARP inhibitors: moving to the adjuvant setting. Nature reviews Clin Oncol.

[70] Marchand JR, Carotti A, Passeri D, Filipponi P, Liscio P, Camaioni E, Pellicciari R, Gioiello A, Macchiarulo A (2014). Investigating the allosteric reverse signalling of PARP inhibitors with microsecond molecular dynamic simulations and fluorescence anisotropy. Biochim Biophys Acta.

[71] Mankouri HW, Huttner D, Hickson ID (2013). How unfinished business from S-phase affects mitosis and beyond. EMBO J.

[72] Crafton SM, Salani R (2016). Beyond chemotherapy: an overview and review of targeted therapy in cervical cancer. Clin Ther.

[73] Deng W, Tsao SW, Lucas JN, Leung CS, Cheung AL (2003). A new method for improving metaphase chromosome spreading. Cytometry A.

