# Supplementary material for: PARP inhibition causes premature loss of cohesion in cancer cells
Source: Oncotarget. 2017 Oct 16;8(61):103931–51. doi: 10.18632/oncotarget.21879 (PMC5732777; doi:10.18632/oncotarget.21879)
Supplement: Supplementary file 1 [file oncotarget-08-103931-s001.pdf]

PARP inhibition causes premature loss of cohesion in cancer cells

SUPPLEMENTARY MATERIALS

Supplementary Table 1: Sequences of siRNAs and qPCR primers used in this study

See Supplementary File 1

Supplementary Table 2: Cell lines used in this study

See Supplementary File 2

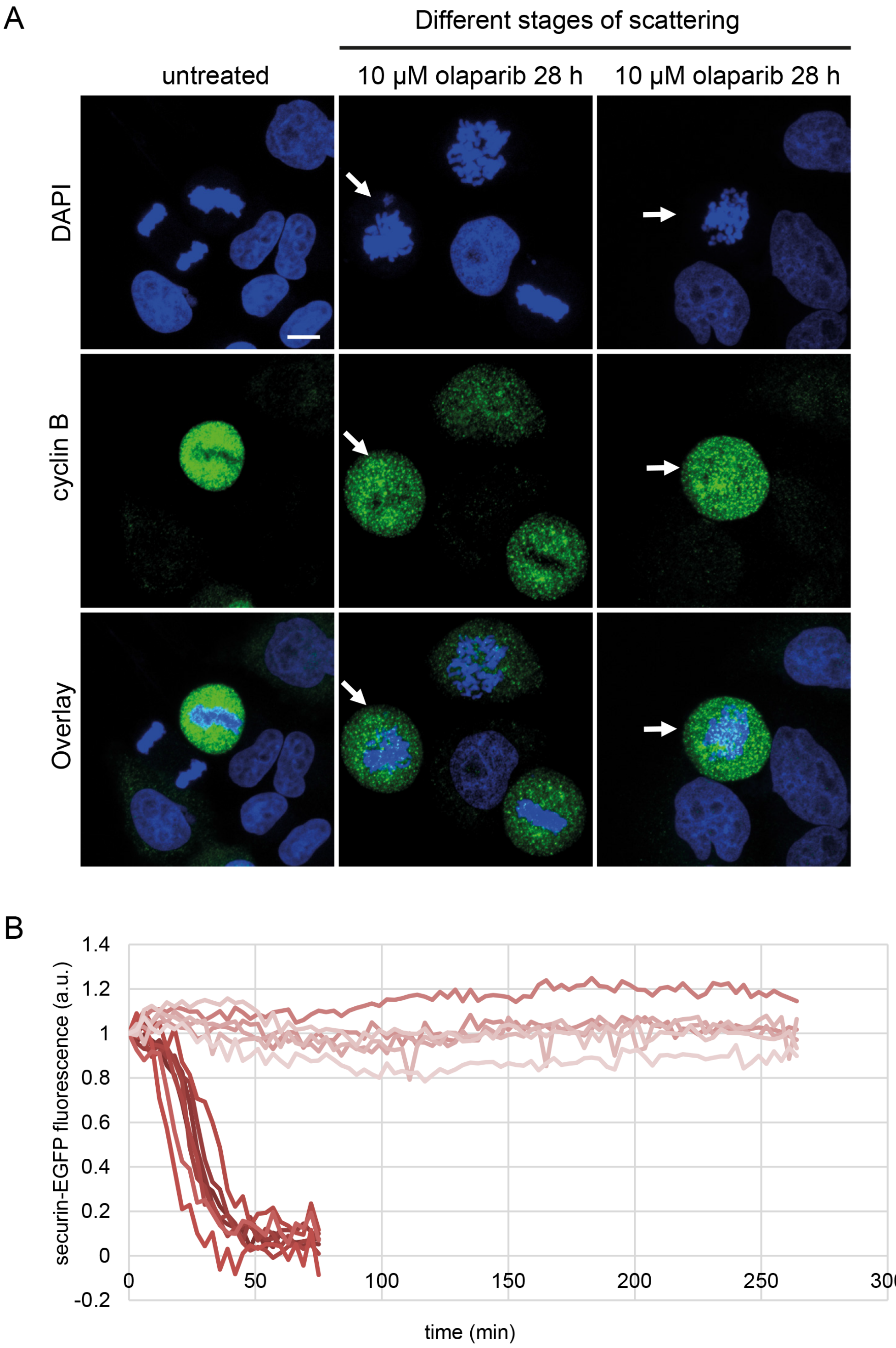

Supplementary Figure 1: Olaparib-treated HeLa H2B-mCherry cells are arrested in metaphase as judged by the lack of cyclin B and securin degradation. **(A)** Immunofluorescence images of cyclin B staining. Cells exhibiting chromosome scattering are indicated with white arrows. Scale bar=10 µm. **(B)** Securin-EGFP fluorescence signal was measured in individual cells (n=6-10 cells per condition). Time zero corresponds to metaphase plate formation. Dark red curves represent cells exiting mitosis and light red represent scattering cells.

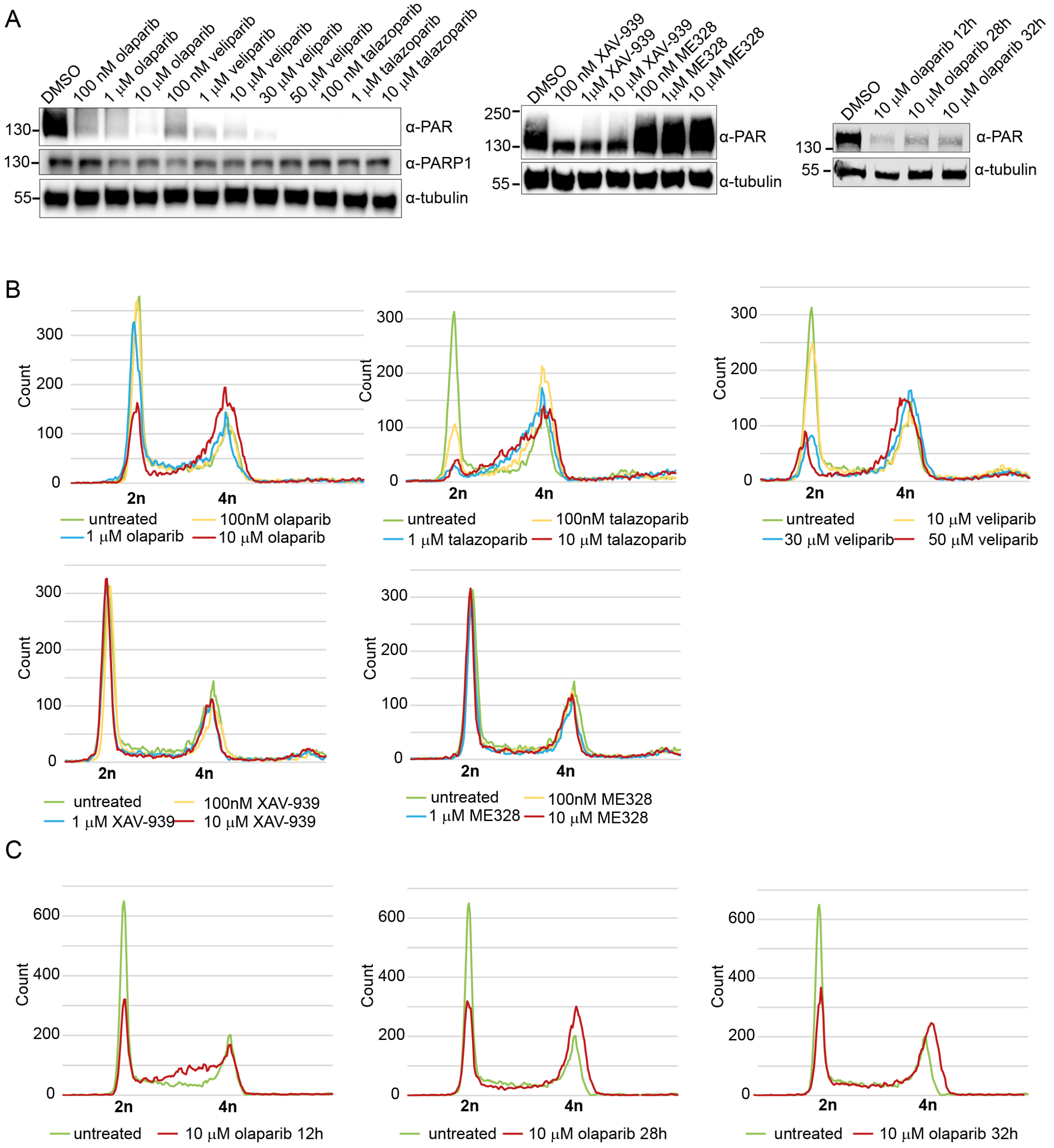

Supplementary Figure 2: The effect of PARP inhibition on (A) PARP1 auto-PARylation and (B,C) cell cycle progression in HeLa. Different concentrations and time points of PARP inhibition were analysed. A 24 h time point was used when examining different inhibitor concentrations.

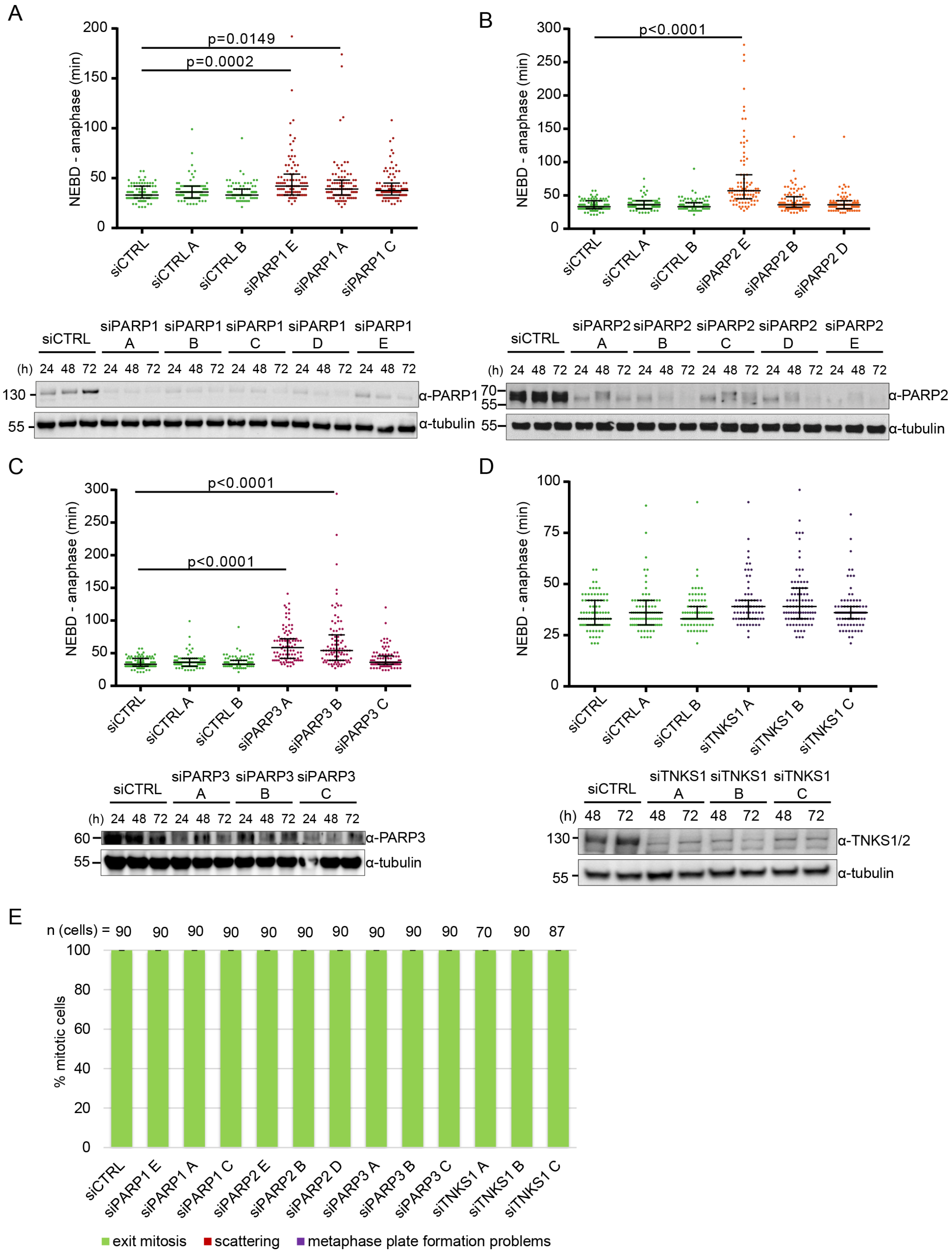

Supplementary Figure 3: RNAi-mediated depletion of PARP1, 2 and 3 does not result in anaphase delay and premature loss of cohesion in HeLa cells. (A-D) Duration of NEBD-anaphase 41-58 h after siRNA transfection. Different siRNAs are indicated with ‘A-E’. Western blot analysis below the graphs shows siRNA efficiency. Tubulin was used as a loading control. **(E)** RNAi treatment does not induce mitotic phenotypes.

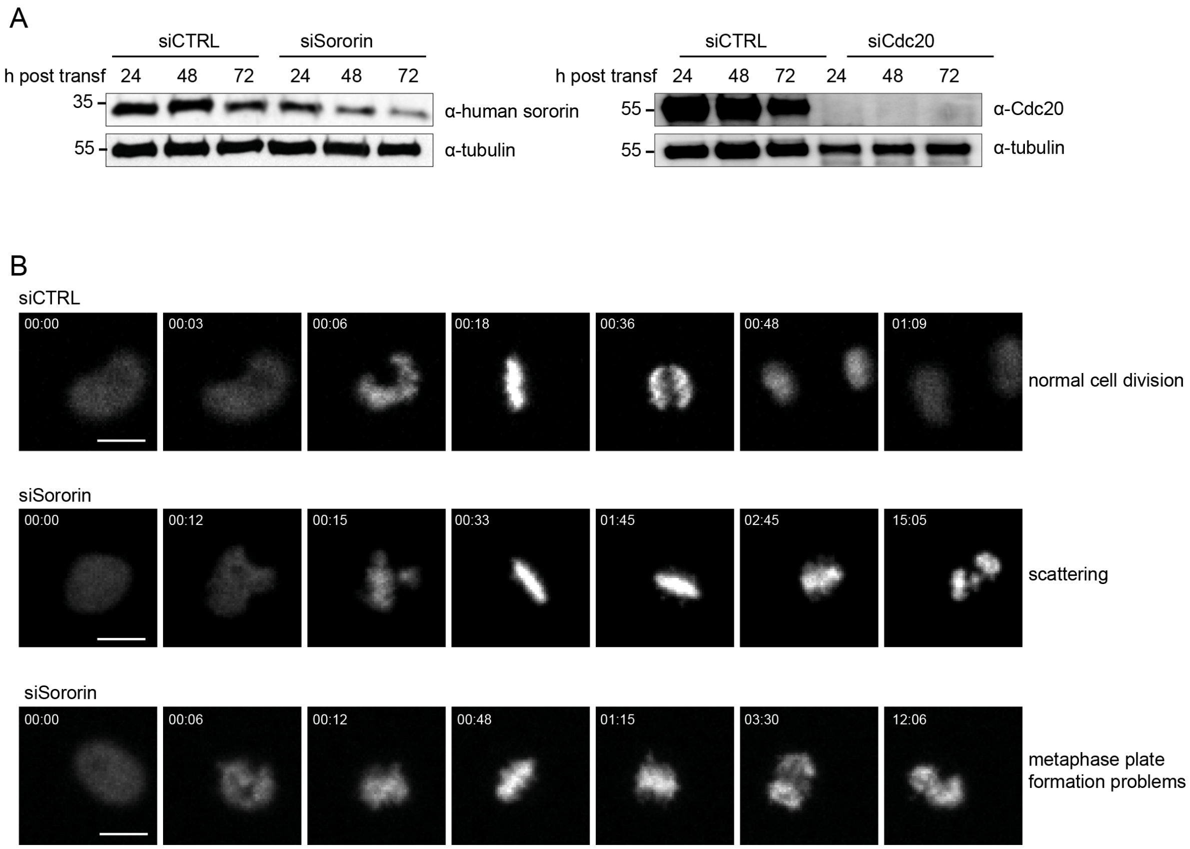

Supplementary Figure 4: Sororin depletion in HeLa H2B-mCherry causes metaphase plate formation problems and scattering. **(A)** Western blot analysis of sororin and Cdc20 RNAi after 24 h. **(B)** Stills from live imaging after sororin RNAi. Scale bar=10 µm.

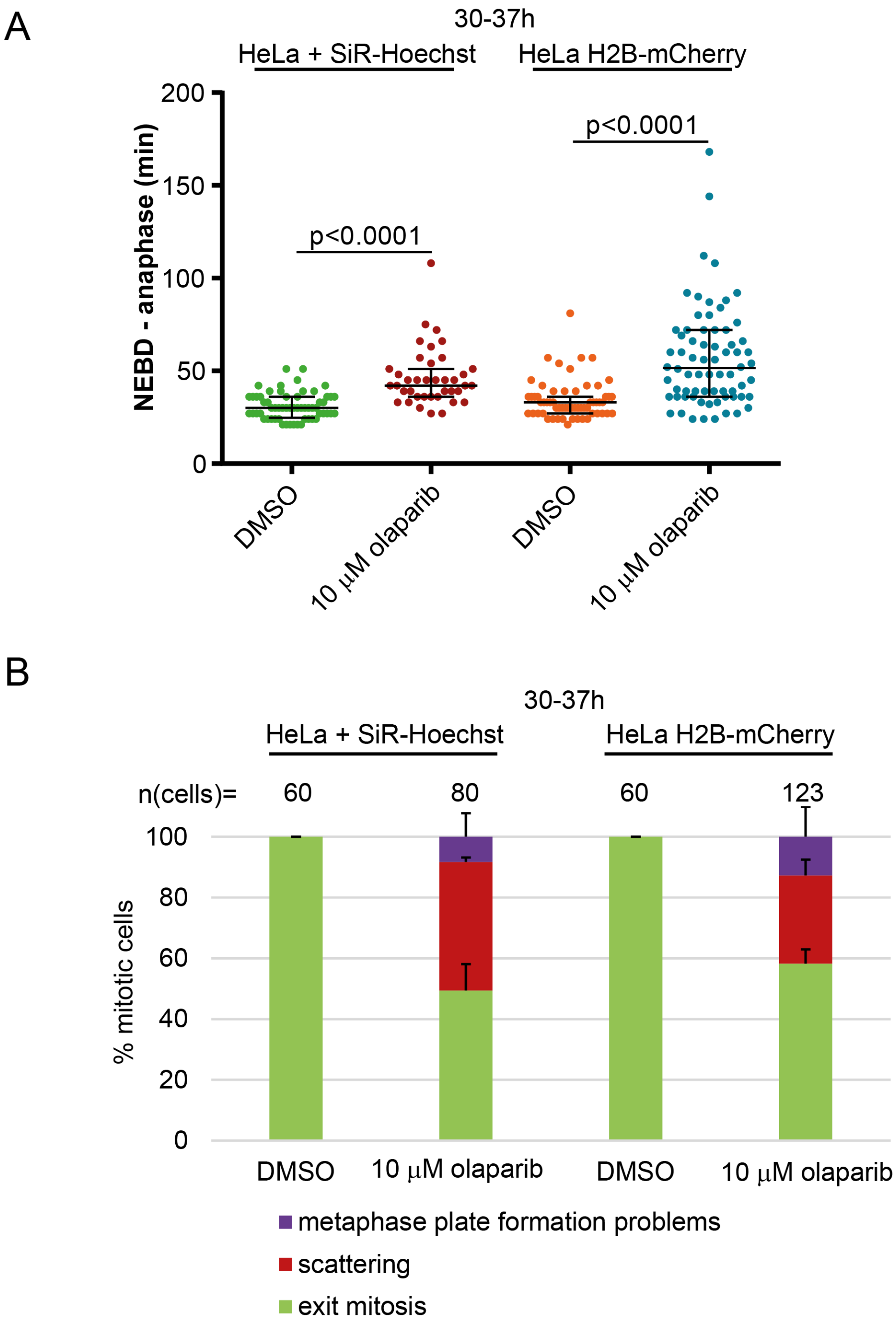

Supplementary Figure 5: SiR-Hoechst-labelled HeLa cells exhibit the same phenotype upon olaparib treatment as HeLa H2B-mCherry cells. **(A)** NEBD-anaphase duration and **(B)** percentage of mitotic phenotypes.

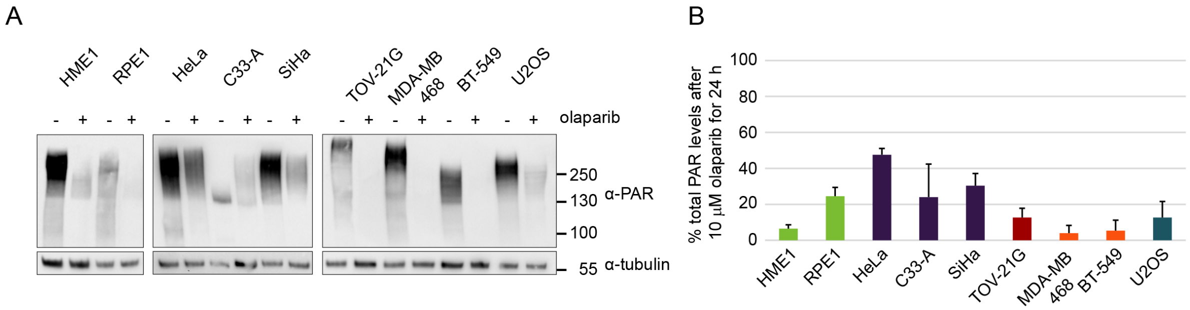

Supplementary Figure 6: The efficiency of PARP inhibition by olaparib in different cell lines. **(A)** Western blot analysis of total PAR levels upon 10 µM olaparib treatment for 24 h in different cell lines. **(B)** Quantification of the percentage of total PAR levels remaining after olaparib treatment. Note that total PAR levels are generated not only by PARP1/2.

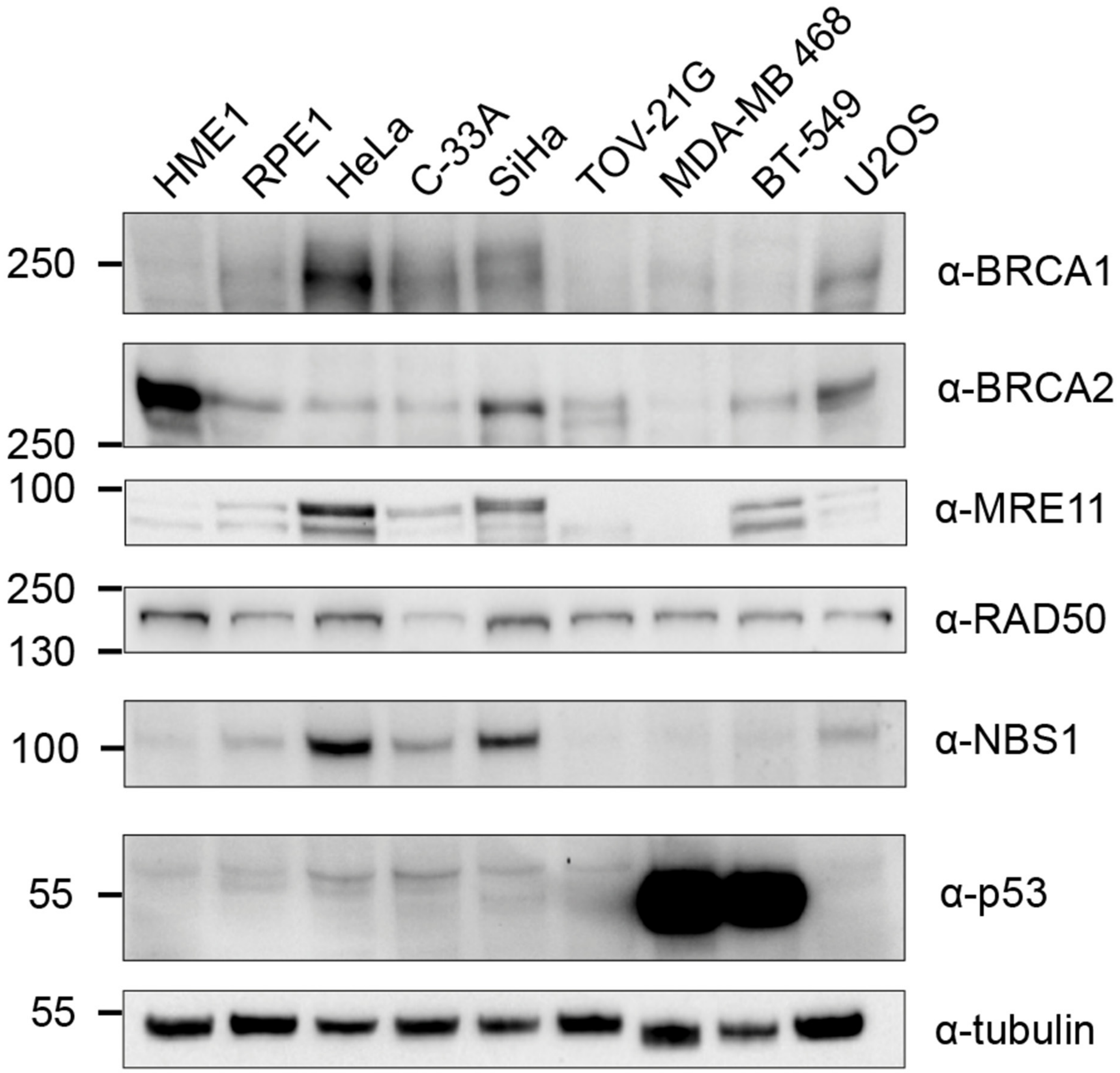

Supplementary Figure 7: Expression levels of P53, BRCA1, BRCA2, MRE11, RAD50 and NBS1 across cell lines examined by Western blotting. Note that specific p53 bands were only detected for MDA-MB 468 and BT-549.

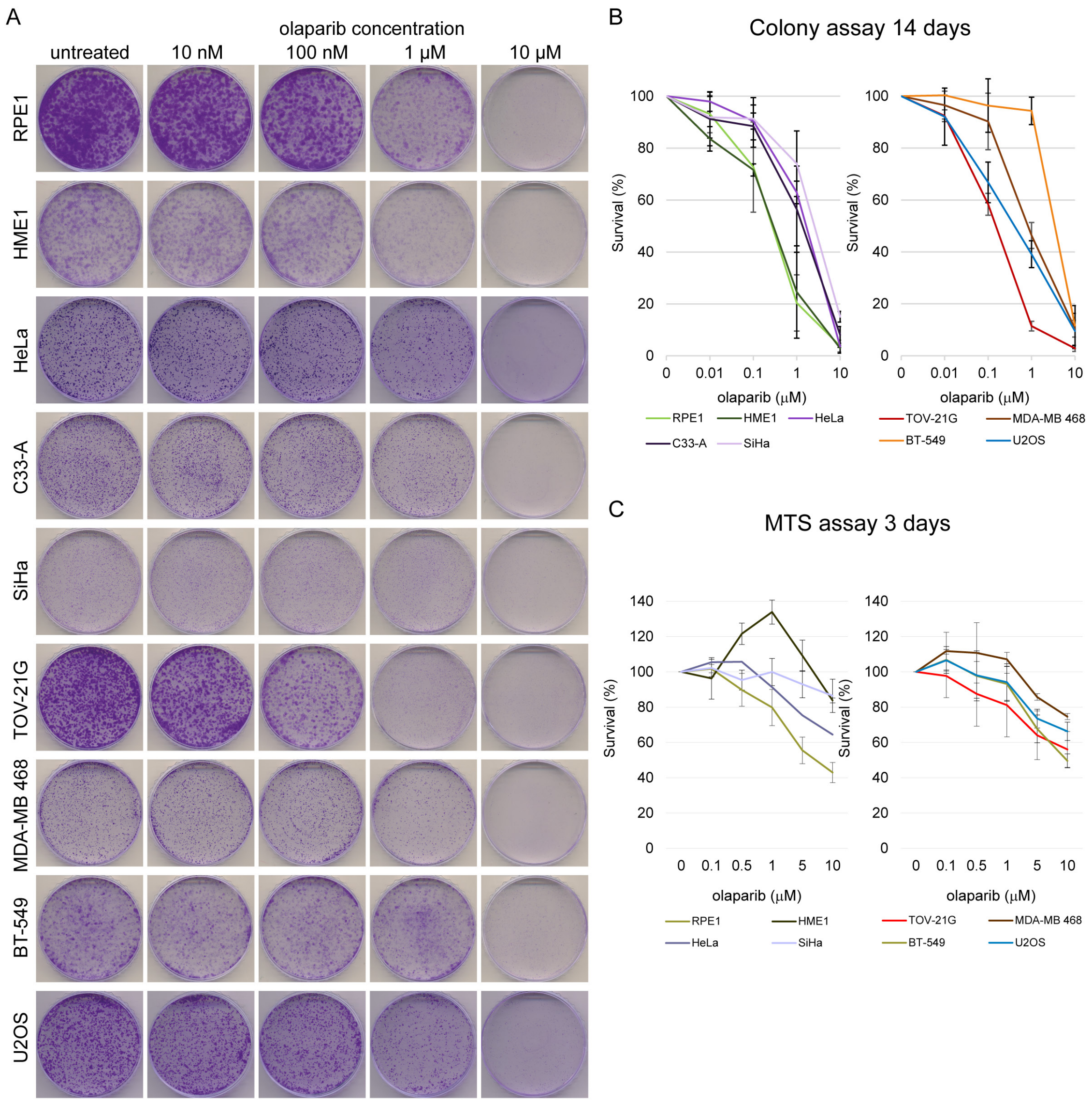

Supplementary Figure 8: Survival of cell lines treated with various concentration of olaparib scored using (A,B) colony formation assay or (C) MTS assay. (A,B) Colonies were stained with crystal violet after 14 days and analysed using Image J plugin 'ColonyArea'. **(C)** CellTiter 96® solution was added after 72 h and the absorbance was measured at 490 nm.

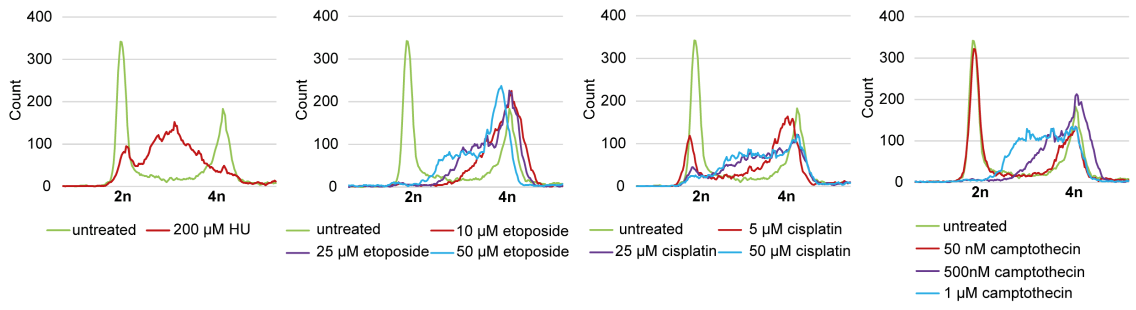

Supplementary Figure 9: Flow cytometry analysis of a 24 h treatment of HeLa H2B-mCherry securin-EGFP cells with indicated concentrations of hydroxyurea, etoposide, cisplatin and camptothecin.
